# Isolation of +2 rare earth metal ions with three anionic carbocyclic rings: bimetallic bis(cyclopentadienyl) reduced arene complexes of La^2+^ and Ce^2+^ are four electron reductants†
†Electronic supplementary information (ESI) available: Experimental and computational details; converged structure data; crystallographic data collection, structure solution, and refinement; and crystallographic data and complete bond distances and angles for compound **2-La**. CCDC 1409845. For ESI and crystallographic data in CIF or other electronic format see DOI: 10.1039/c5sc02486b


**DOI:** 10.1039/c5sc02486b

**Published:** 2015-09-21

**Authors:** Christopher M. Kotyk, Megan E. Fieser, Chad T. Palumbo, Joseph W. Ziller, Lucy E. Darago, Jeffrey R. Long, Filipp Furche, William J. Evans

**Affiliations:** a Department of Chemistry , University of California , Irvine , California 92697 , USA . Email: wevans@uci.edu ; Email: filipp.furche@uci.edu ; Fax: +1-949-824-2210 ; Tel: +1-949-824-5174; b Department of Chemistry , University of California , Berkeley , California 94720 , USA . Email: jrlong@berkeley.edu

## Abstract

A new option for stabilizing unusual Ln^2+^ ions has been identified in the reaction of Cp′_3_Ln, **1-Ln** (Ln = La, Ce; Cp′ = C_5_H_4_SiMe_3_), with potassium graphite (KC_8_) in benzene in the presence of 2.2.2-cryptand. This generates [K(2.2.2-cryptand)]_2_[(Cp′_2_Ln)_2_(μ-η^6^:η^6^-C_6_H_6_)], **2-Ln**, complexes that contain La and Ce in the formal +2 oxidation state. These complexes expand the range of coordination environments known for these ions beyond the previously established examples, (Cp′′_3_Ln)^1–^ and (Cp′_3_Ln)^1–^ (Cp′′ = C_5_H_3_(SiMe_3_)_2_-1,3), and generalize the viability of using three anionic carbocyclic rings to stabilize highly reactive Ln^2+^ ions. In **2-Ln**, a non-planar bridging (C_6_H_6_)^2–^ ligand shared between two metals takes the place of a cyclopentadienyl ligand in (Cp′_3_Ln)^1–^. The intensely colored (*ε* = ∼8000 M^–1^ cm^–1^) **2-Ln** complexes react as four electron reductants with two equiv. of naphthalene to produce two equiv. of the reduced naphthalenide complex, [K(2.2.2-cryptand)][Cp′_2_Ln(η^4^-C_10_H_8_)].

## Abstract 2

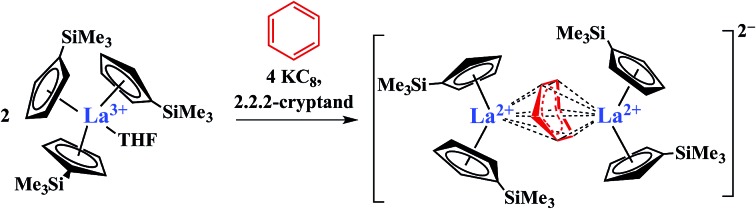
A new option for stabilizing unusual Ln^2+^ ions has been identified in the reaction of (C_5_H_4_SiMe_3_)_3_Ln (Ln = La, Ce) with potassium graphite and 2.2.2-cryptand in benzene.

## 

One of the most fundamental aspects of any element is the number of oxidation states accessible to it. Since this defines the range of chemistry possible with the element, the limits of oxidation states for each element have been heavily probed for decades and are well established. Surprisingly, in the last few years, a new oxidation state has been discovered for nine elements in the rare earth series.^1^–^4^

The discovery of the nine new Ln^2+^ ions required,^1^–^4^ in each case, a coordination environment composed of three cyclopentadienyl rings, specifically (Cp′′_3_)^3–^ or (Cp′_3_)^3–^ (Cp′′ = C_5_H_3_(SiMe_3_)_2_-1,3; Cp′ = C_5_H_4_SiMe_3_), Scheme 1. In this tris(cyclopentadienyl) ligand field, it was found that reduction of a 4f^*n*^ Ln^3+^ ion added an electron, not to the 4f valence orbitals to make a 4f^*n*+1^ Ln^2+^ ion, but to an orbital with a higher principal quantum number, a 5d_*z*^2^_ orbital, to give Ln^2+^ ions best described by 4f^*n*^5d^1^ electron configurations. This was rationalized by the fact that the d_*z*^2^_ orbital is the lowest energy d orbital in a tris(cyclopentadienyl) coordination environment complex.^1^–^11^

**Scheme 1:**
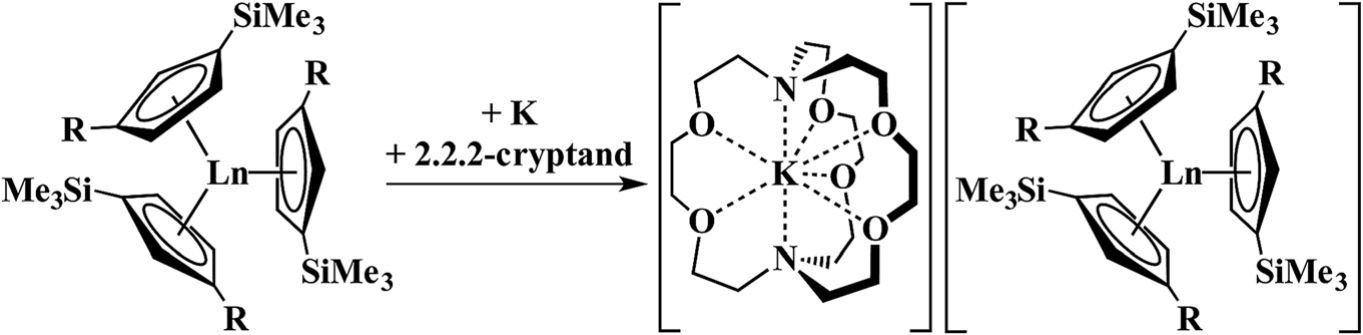
Reduction of Cp′′_3_Ln (R = SiMe_3_; Ln = La) and Cp′_3_Ln (R = H; Ln = Y, La, Ce, Pr, Nd, Sm, Gd, Tb, Dy, Ho, Er, Tm, Lu) to form Ln^2+^ complexes.^1^–^4^

It was of interest to determine if these Ln^2+^ ions could be isolated in other coordination environments to examine the general accessibility of these new oxidation states since Ln^2+^ ions have proven to be broadly useful reductants as in the use of Sm^2+^ in organic chemistry.^12^,^13^ Crystallographic evidence that Ln^2+^ complexes could be made with an anionic reduced benzene ligand was previously suggested by Lappert *et al.*^14^,^15^ as shown in Scheme 2.

**Scheme 2:**
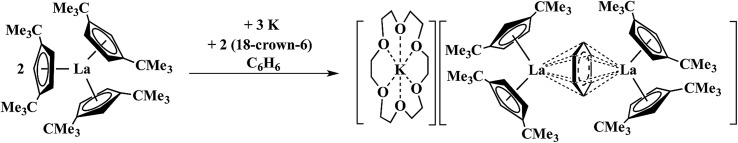
Reaction of Cp^tt^_3_La Cp^tt^ = C_5_H_3_(CMe_3_)_2_]-1,3 with 1.5 equiv. of K in benzene to form a (C_6_H_6_)^1–^ complex.^14^

Reduction of Cp^tt^_3_La (Cp^tt^ = C_5_H_3_(CMe_3_)_2_-1,3) with 1.5 equiv. of potassium in the presence of benzene gave a compound that was postulated to be a La^2+^ complex of a (C_6_H_6_)^1–^ bridging ligand rather than a Ln^3+^ complex of (C_6_H_6_)^3–^. With the silyl analogs, Cp′′_3_Ln (Ln = La, Ce), in toluene, similar reactions to form (C_6_H_5_CH_3_)^1–^ complexes were reported.^15^ The known difficulty in assigning oxidation states in bridging arene systems^16^–^25^ complicated the assignments until unambiguous examples of La^2+^ and Ce^2+^ were found *via*Scheme 1.^1^

In a small variation of Scheme 2, reactions involving excess K instead of 1.5 equiv. per Ln led to the formation of Ln^3+^ products, rather than Ln^2+^ complexes and reduction of benzene to (C_6_H_6_)^2–^ rather than (C_6_H_6_)^1–^ in the compounds, [K(18-crown-6)][(C_6_H_6_)LnCp′′_2_] (Ln = La, Ce, Pr, Nd), Scheme 3.^26^,^27^ In a further variation with (C_5_H_4_SiMe_2_CMe_3_)_3_Ln precursors in toluene, Ln^3+^ hydride products were found.^12^ All of these reactions were postulated to involve Ln^2+^ intermediates.

**Scheme 3:**
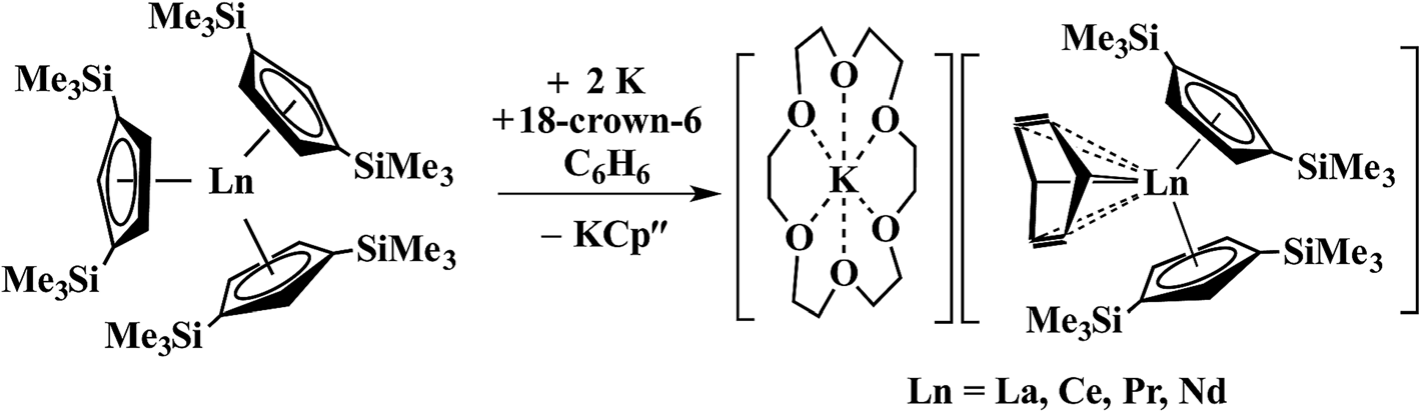
Reduction of benzene to (C_6_H_6_)^2–^ by Cp′′_3_Ln/K.^26^,^27^

Further variability in this arene rare earth reduction chemistry was subsequently observed with Cp′ ligands in reactions of crystallographically characterized Ln^2+^ complexes, [K(2.2.2-cryptand)][Cp′_3_Ln], **3-Ln** (Ln = Y, La, Ce, Dy).^3^,^4^ These compounds reduce naphthalene (–2.50 V *vs.* SCE^19^) and biphenyl (–2.69 V *vs.* SCE^19^), but were not observed to reduce benzene (–3.43 V *vs.* SCE^19^). These reactions differed from Schemes 2 and 3 not only in the Cp′ ligand, but also in that they started with bona fide Ln^2+^ precursors and not a combination of a Ln^3+^ precursor and potassium that could form a Ln^2+^ intermediate.

In light of these results, it was of interest to see how the Cp′ complexes would behave in reactions analogous to Schemes 2 and 3. This ligand has given yet another variation on benzene reduction and has led to a new series of rare earth complexes in the formal +2 oxidation state. These results show the generality of using three anionic carbocyclic ligands to stabilize Ln^2+^ and provide a new type of four-electron reductant.

Reactions of solutions of Cp′_3_Ln, **1-Ln** (Ln = La, Ce), in benzene with 2 equiv. of potassium–graphite (KC_8_) in the presence of 2.2.2-cryptand produce thick black precipitates. After stirring at room temperature for 4 h, extraction with THF followed by centrifugation to remove graphite produces deep purple solutions from which deep purple crystals of [K(2.2.2-cryptand)]_2_[(Cp′_2_Ln)_2_(μ-η^6^:η^6^-C_6_H_6_)], **2-Ln**, were isolated. Single crystals were grown from THF and identified by X-ray diffraction, although only **2-La** gave good metrical data, Fig. 1. The byproduct [K(2.2.2-cryptand)][Cp′] was identified by ^1^H NMR spectroscopy.

**Fig. 1:**
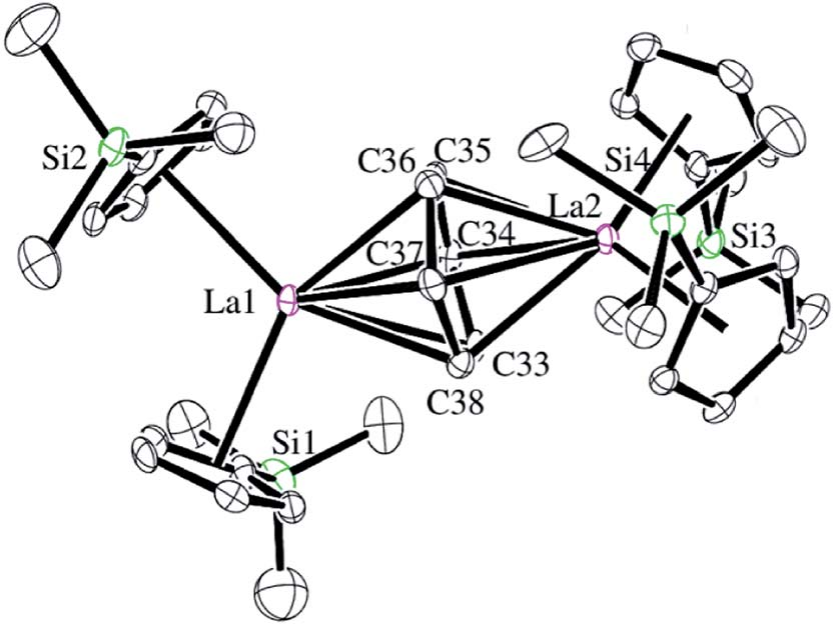
Molecular structure of the anion of [K(2.2.2-cryptand)]_2_[(Cp′_2_La)_2_(μ-η^6^:η^6^-C_6_H_6_)], **2-La**. Thermal ellipsoids are drawn at the 50% probability level. Hydrogen atoms and co-crystallized benzene and THF were omitted for clarity.

The reaction formally involves the addition of four equiv. of KC_8_ to two equiv. of Cp′_3_Ln with loss of one (Cp′)^1–^ anion per metal complex as [K(2.2.2-cryptand)][Cp′] and formation of a reduced benzene ligand bridging two bis(cyclopentadienyl) metal units, Scheme 4. The [(Cp′_2_Ln)_2_(μ-η^6^:η^6^-C_6_H_6_)]^2–^ product could be described by two extreme forms as either two Ln^2+^ ions and a (C_6_H_6_)^2–^ dianion or two Ln^3+^ ions and a (C_6_H_6_)^4–^ tetraanion.

**Scheme 4:**
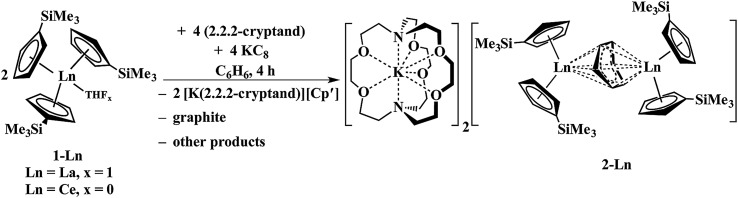
Reduction of benzene by Cp′_3_Ln/KC_8_ to form [K(2.2.2-cryptand)]_2_[(Cp′_2_La)_2_(μ-η^6^:η^6^-C_6_H_6_)], **2-Ln** (Ln = La, Ce).

^1^H NMR analysis of **2-La** shows multiple resonances at chemical shifts typical of aromatic Cp′ protons (5.5 to 6.0 ppm), known 2.2.2-cryptand resonances, and peaks in the 0.0 to 0.5 ppm range typical of trimethylsilyl protons. A peak at 2.03 ppm in the ^1^H NMR spectrum was assigned to the reduced arene since it was missing in the analogous reaction using C_6_D_6_. The product of the reaction described in Scheme 4 with C_6_D_6_ gives a product with a resonance at 2.03 ppm in the ^2^H NMR spectrum.

The compound **2-La** exhibits diamagnetic behavior, with an extremely small (∼10^–2^ emu K mol^–1^) magnetic susceptibility across the temperature range 2–300 K. The gradual increase in *χ*_M_*T* with increasing temperature is likely due to a very small population of a triplet excited state or field-induced mixing with a triplet excited state. While the diamagnetism of **2-La** may at first seem indicative of a La^3+^/(C_6_H_6_)^4–^ electron configuration, we instead propose, in agreement with the crystallographic analysis, UV-vis spectroscopy, and electronic structure calculations, that extremely strong magnetic exchange coupling between the two La^2+^ (d^1^) centers and the (C_6_H_6_)^2–^ diradical generates a well-isolated singlet ground state for **2-La**. Indeed, DFT calculations reveal the energy splitting between the ground state and first excited triplet state to be 10500 cm^–1^, well over an order of magnitude higher than the thermal energy at 300 K. The magnetic data could also be reasonably well-simulated using the Hamiltonian *Ĥ* = –2*J*_La–C_6_H_6__ (*ŝ*_La1_ × *ŝ*_C_6_H_6__+ *ŝ*_La2_ × *ŝ*_C_6_H_6__), where *J*_La–C_6_H_6__ represents the coupling between the *S* = 1/2 La^2+^ centers and the *S* = 1 (C_6_H_6_)^2–^ bridging unit, along with a temperature-independent paramagnetism contribution, *χ*_TIP_. The best simulation was achieved using the values *J*_La–C_6_H_6__ = –497 cm^–1^ and *χ*_TIP_ = 0.000057 emu mol^–1^ (Fig. 5). Thus, the diamagnetism observed across the measured temperature range is also consistent with an assignment of La^2+^/(C_6_H_6_)^2–^ together with a pairwise exchange constant of *J*_La–C_6_H_6__ < |–500 cm^–1^|.

The variable-temperature magnetic susceptibility of **2-Ce** alone also does not enable a definitive assignment of the oxidation states present. At 300 K under an applied magnetic field of 0.1 T, **2-Ce** exhibits a *χ*_M_*T* product of 1.78 emu K mol^–1^, which then drops to 1.36 emu K mol^–1^ under an increased applied magnetic field of 1 T. The field dependence of **2-Ce** is due to temperature-independent paramagnetism, which has been previously observed for both Ce^2+^ and Ce^3+^ compounds.^28^,^29^ The expected *χ*_M_*T* value at 300 K for a Ce^3+^/(C_6_H_6_)^4–^ configuration is 1.60 emu K mol^–1^. The situation for a Ce^2+^-based electronic configuration is much more complicated, as even for a simple mononuclear Ce^2+^ complex the room temperature *χ*_M_*T* value falls in between those expected for “uncoupled” and “coupled” 4f^1^5d^1^ configurations.^29^ The LS coupling schemes for 4f^*n*^5d^1^ configurations detailed in ref. 29 can be used, in tandem with the assumption that at 300 K the *S* = 1 (C_6_H_6_)^2–^ moiety is not magnetically coupled to the Ce^2+^ ions, to predict room temperature *χ*_M_*T* values for **2-Ce**. The calculated *χ*_M_*T* products at 300 K for **2-Ce** assuming “uncoupled” and “coupled” 4f^1^5d^1^ configurations plus an isolated *S* = 1 center are 3.36 emu K mol^–1^ and 1.66 emu K mol^–1^, respectively. Clearly the former value does not match the *χ*_M_*T* data observed for **2-Ce**. However, the latter value for the “coupled” scheme is quite close to the experimental *χ*_M_*T* product for **2-Ce**. If magnetic coupling between the Ce^2+^ spins and the (C_6_H_6_)^2–^ diradical is strong, as proposed above for **2-La**, the expected *χ*_M_*T* product at 300 K for **2-Ce** will be even lower than 1.66 emu K mol^–1^, and thus closer to the observed value of 1.36 emu K mol^–1^ at 1 T. If extremely strong d–π* magnetic coupling is present in **2-Ce**, the resulting magnetic behavior could even appear much like that of a standard dinuclear Ce^3+^ (4f^1^) molecule. Therefore, discerning between the possible electronic configurations for **2-Ce**, Ce^3+^/(C_6_H_6_)^4–^ or Ce^2+^/(C_6_H_6_)^2–^, is not possible from the magnetic data alone.

The structural parameters for [K(2.2.2-cryptand)]_2_[(Cp′_2_La)_2_(μ-η^6^:η^6^-C_6_H_6_)], **2-La**, are summarized in Table 1. The 1.446(6)–1.459(6) Å C–C bonds of the C_6_ unit in the solid-state structure of **2-La** are longer than the bond lengths in free benzene, which has an average C–C bond length of 1.397(9) Å.^30^ This is consistent with reduction of C_6_H_6_. The C_6_H_6_ moiety is not planar and has a dihedral angle of 11° between the planes defined by C34–C37 and C33, C34, C37, C38. This is more consistent with (C_6_H_6_)^2–^ than (C_6_H_6_)^4–^, since (C_6_H_6_)^4–^ is reported to be planar.^19^ Interestingly, the 2.690 Å La–(Cp′ ring centroid) distances are significantly longer than those of either the La^3+^ complex, Cp′_3_La, 2.559 Å,^31^ or the La^2+^ complex, [K(2.2.2-cryptand)][Cp′_3_La], **2-La**, 2.586 Å.^4^ Traditionally, bond distances in 4f^*n*+1^ Ln^2+^ complexes are 0.1–0.2 Å larger than those of 4f^*n*^ Ln^3+^ complexes, but bond distances for 4f^*n*^5d^1^ complexes of the recently discovered Ln^2+^ ion complexes are only 0.02–0.03 Å longer.^4^ A referee has noted that the La1–C34 and La–C37 distances, the shortest for La1–C(C_6_H_6_), correlate with the longest La2–C(C_6_H_6_) distances, La2–C34 and La2–C37. Similarly, the shortest La2–C(C_6_H_6_) distances, La2–C33 and La2–C36, correlate with the longest La1–C(C_6_H_6_) distances, La1–C33 and La1–C36.

**Table 1:** Selected bond distances (Å) and angles (°) for [K(2.2.2-cryptand)]_2_[(Cp′_2_La)_2_(μ-η^6^:η^6^-C_6_H_6_)], **2-La**.

**2-La**					
La1–Cnt1^*a*^	2.692	La1–C33	2.770(4)	Cnt1–La1–Cnt2^*a*^	111.5
La1–Cnt2^*a*^	2.681	La1–C34	2.639(4)	Cnt1–La1–Cnt5^*a*^ ^,^^*b*^	125.1
La2–Cnt3^*a*^	2.687	La1–C35	2.694(5)	Cnt2–La1–Cnt5^*a*^ ^,^^*b*^	123.4
La2–Cnt4^*a*^	2.709	La1–C36	2.777(5)	Cnt3–La2–Cnt4^*a*^	112.6
La1–Cnt5^*b*^	2.278	La1–C37	2.641(5)	Cnt3–La2–Cnt5^*a*^	123.0
La2–Cnt5^*b*^	2.273	La1–C38	2.680(4)	Cnt4–La2–Cnt5^*a*^ ^,^^*b*^	124.5
C33–C34	1.457(6)	La2–C33	2.637(5)	Pln1–Pln2^*c*^	11.0
C34–C35	1.448(7)	La2–C34	2.776(5)		
C35–C36	1.446(6)	La2–C35	2.683(4)		
C36–C37	1.459(6)	La2–C36	2.635(5)		
C37–C38	1.456(6)	La2–C37	2.766(4)		
C38–C33	1.454(6)	La2–C38	2.676(4)		

^*a*^Cnt1, Cnt2, Cnt3, and Cnt4 are the centroids of the Cp′ groups.

^*b*^Cnt5 is the centroid of the C6 unit labeled C33–C38.

^*c*^Pln1 and Pln2 are the planes of (C33–C36) and (C33, C36, C37, C38), respectively.

The UV-vis spectra for **2-Ln** are shown in Fig. 2 and compared to the Ln^2+^ complexes, [K(2.2.2-cryptand)][Cp′_3_Ln], **3-Ln**.^3^,^4^ The spectra of **2-Ln** are similar to those of **3-Ln**, but the extinction coefficients are much higher: ∼8000 M^–1^ cm^–1^ in the high-energy visible region. This is particularly unusual since the 1000–2000 M^–1^ cm^–1^ extinction coefficients for **3-Ln** are already much higher than those of analogous complexes of traditional 4f^*n*^ Ln^2+^ complexes of **3-Ln** which are all lower than 900 M^–1^ cm^–1^.^4^

**Fig. 2:**
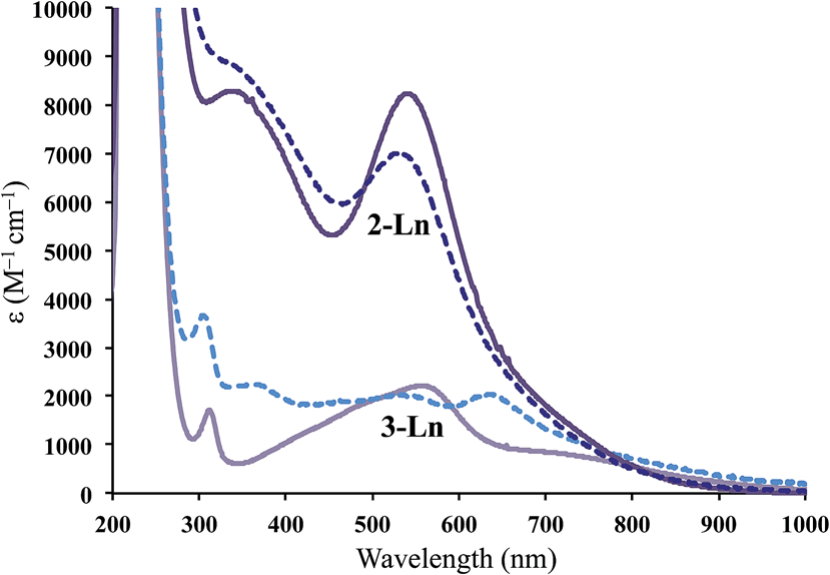
Experimental UV-vis spectra of [K(2.2.2-cryptand)]_2_[(Cp′_2_Ln)_2_(μ-η^6^:η^6^-C_6_H_6_)], **2-Ln** (dark), and [K(2.2.2-cryptand)][Cp′_3_Ln], **3-Ln** (light), in THF (1 mM) at 298 K (Ln = La, solid; Ln = Ce, dotted).

DFT calculations were used to examine the energies of the possible electron configurations of **2-La**. All calculations were performed using the Turbomole quantum chemistry software^32^ and the TPSSh functional (further details are provided in the ESI†).^33^ Calculations on [(Cp′_2_Ln)_2_(μ-η^6^:η^6^-C_6_H_6_)]^2–^ found an energy minimum corresponding to a diamagnetic singlet ground state that had metrical parameters that matched the crystal data better than any other electronic configuration. The calculations also showed long La–(Cp′ ring centroid) distances, similar to those found experimentally. However, the La–C(C_6_H_6_) bond lengths in the calculated minimum were 0.05 Å longer than those in the crystal structure. Single-point energy calculations indicate the triplet and quintet states are 30 kcal mol^–1^ (10500 cm^–1^) and 62 kcal mol^–1^ (21800 cm^–1^) higher than the singlet ground state, respectively. When a geometry optimization of the quintet state was performed, the optimized structure had only three carbon atoms of the bridging C_6_H_6_ ligand coordinated to one metal and the other three carbon atoms coordinated to the other metal. This large difference from the experimental structure indicated that this quintet state is not a viable description of **2-La**.

The two highest occupied molecular orbitals (HOMO and HOMO–1) of the singlet state show significant mixing between the metal orbitals and the π* orbitals of the C_6_H_6_ ring, Fig. 3. Mulliken population analyses (MPA)^34^ of the HOMO suggest that 61% of the orbital is localized on π* orbitals of the C_6_H_6_ ring with 39% involved with the two metals centers. For HOMO–1, the orbital is 64% on the ring and 36% on the two metals. This is less than that for the HOMO of the Ln^2+^ ions in (Cp′_3_Ln)^1–^ (from calculations with identical computational methods), which is often >70% metal-based, depending on the lanthanide, and is primarily a d_*z*^2^_ orbital.

**Fig. 3:**
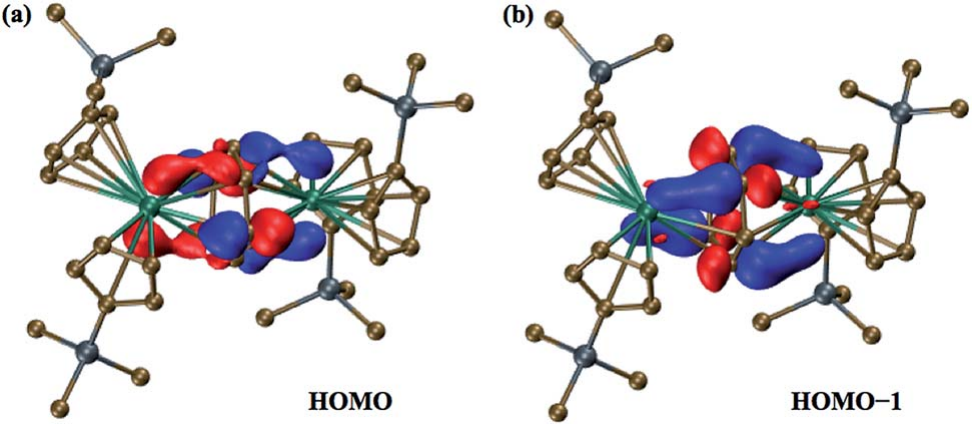
Molecular orbital plots of (a) the 183a orbital (HOMO) and (b) the 182a orbital (HOMO–1) of the dianion in **2-La**, using a contour value of 0.05.

Natural population analysis (NPA)^35^ of the dianion in **2-La** suggests that each La center has approximately 1.4 5d electrons, Table 2, which is more than the 1.2 electrons and 0.9 electrons found in calculations on (Cp′_3_La)^1–^ and Cp′_3_La, **1-La**, respectively.^4^ Since there is more electron density on each La center in **2-La** than even that for the La^2+^ complex, [K(2.2.2-cryptand)][Cp′_3_La], **3-La**,^4^ these calculations indicate that the description involving two Ln^2+^ ions and a (C_6_H_6_)^2–^ dianion is the most accurate of the two extreme structures considered above. The mixed arene/d orbital character of the HOMO and HOMO–1 orbitals suggests that it is not a rigid requirement for isolation of Ln^2+^ complexes of metals such as lanthanum and cerium to have ligand fields that provide a low lying d_*z*^2^_ orbital.

**Table 2:** MPA and NPA analysis of the dianion in **2-La**. The % metal character indicates the total metal contribution to the molecular orbital and the % d character indicates how much of the total orbital comes directly from the metal d orbitals.

Metal center	(HOMO–1) MPA		(HOMO) MPA		NPA total density (5d orbital)
	% metal	% d	% metal	% d	
La1	18	13	20	18	1.4
La2	18	13	19	18	1.4

Time dependent DFT (TDDFT)^36^ calculations were performed to simulate the UV-vis spectrum of **2-La**. The simulated spectrum of the singlet state gave the best match to the experimental spectrum as shown in Fig. 4. The predicted UV-vis spectrum contains three broad absorptions, one of which matches the low energy absorption in the experimental spectrum, while the combination of the other two fit under the experimentally-determined high-energy absorption. The excitations between 300 and 1000 nm are comprised of excitations from the HOMO and HOMO–1 orbitals shown in Fig. 3. The lowest energy transitions (>440 nm) involve transitions to primarily metal-based orbitals with s and/or d character, while the highest energy transitions (<440 nm) involve transitions to primarily ligand-based orbitals.

**Fig. 4:**
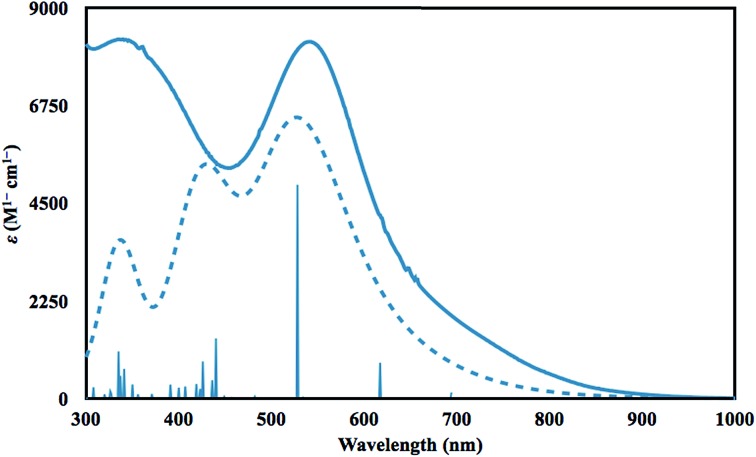
Experimental (solid) and calculated (dotted) UV-vis spectra of **2-La** in THF at 298 K, with pertinent theoretical excitations shown as vertical lines and theoretical extinction coefficients scaled down by a factor of 4.7.

**Fig. 5:**
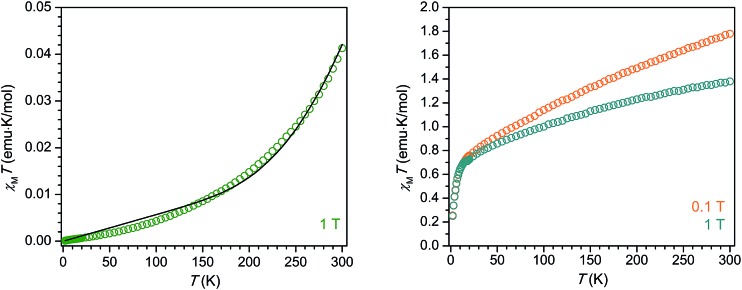
Variable-temperature magnetic susceptibility data collected for **2-La** under *H*_dc_ = 1 T (green). Simulation of the data using the model described in the text is represented by a solid black line. Variable-temperature magnetic susceptibility data collected for **2-Ce** under *H*_dc_ = 0.1 T (orange) and 1 T (blue).

The reductive reactivity of **2-Ln** was probed by examining the reaction with naphthalene. Two equiv. of naphthalene are reduced by four electrons to produce 2 equiv. of [K(2.2.2-cryptand)][Cp′_2_Ln(η^4^-C_10_H_8_)], **4-Ln** (Ln = La, Ce), Scheme 5.^37^ The four electron reduction is consistent with the presence of two Ln^2+^ ions and a (C_6_H_6_)^2–^ dianion, but does not provide definitive evidence on this because a (C_6_H_6_)^4–^ anion would also be a 4-electron reductant. This reaction does provide a clean route to **4-Ln**, which are originally made from **3-Ln** in a reaction that has an inseparable byproduct, [K(2.2.2-cryptand)][Cp′_4_Ln] (Ln = Y, La) that required **4-La** to be separated *a la Pasteur*.^37^

**Scheme 5:**
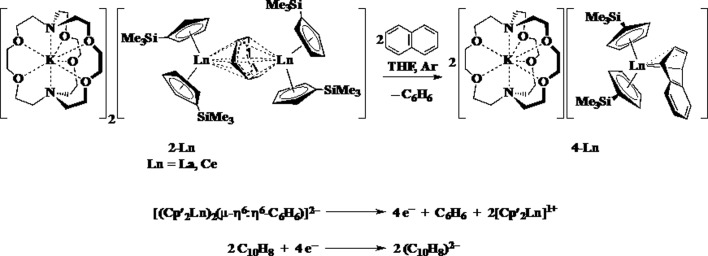
Reduction of naphthalene by **2-Ln** to produce **4-Ln**.

Complex **2-Ln** does not appear to react with N_2_, but it does reduce 1,3,5,7-cyclooctatetraene in a reaction that is more complicated than Scheme 5. [K(2.2.2-crypt)][(C_8_H_8_)_2_La] was identified as the main product by independent synthesis from K[(C_8_H_8_)_2_La] made in 1973.^38^ Attempts to reduce **2-Ln** further did not give isolable organometallic products.

In summary, a new type of rare earth compound has been identified that is best described as a bimetallic complex of two Ln^2+^ ions bridged by (C_6_H_6_)^2–^. This result constitutes an intriguing variation of the arene reduction reactions in Schemes 2 and 3 in which complexes are found with either (C_6_H_6_)^1–^ and two Ln^2+^ ions or with (C_6_H_6_)^2–^ and one Ln^3+^ ion. Clearly, small variations in these reactions and in the substituents on the cyclopentadienyl rings can have a significant effect on the product isolated in the tris(cyclopentadienyl)rare earth/alkali metal reductions of arenes.

The isolation of **2-Ln** demonstrates the generality of isolating Ln^2+^ ions with three anionic carbocyclic rings beyond the (Cp′′_3_)^3–^ and (Cp′_3_)^3–^ coordination environments. In **2-Ln**, a (C_6_H_6_)^2–^ dianion shared between two metals takes the place of a cyclopentadienyl anion in the monometallic Ln^2+^ systems.^1^–^3^ This is not a common substitution in organometallic chemistry since arene anions are only formed under highly reducing conditions. DFT studies on **2-La** show that this heteroleptic three ring ligand system differs from the tris(cyclopentadienyl) complexes in that the HOMOs have much more ligand character than in the (Cp′′_3_)^3–^ and (Cp′_3_)^3–^-ligated complexes, in which the HOMOs are primarily d_*z*^2^_. These results suggest that other variations of three carbocyclic rings with orbital character beyond that found in tris(cyclopentadienyl) ligand environments could also stabilize unusual Ln^2+^ ions.

It is important to note that **2-La** displays some unexpected properties, *i.e.* the long La–(Cp′ ring centroid) distances in the crystal structure, the long La–C(C_6_H_6_) distances in the DFT calculations, and the extremely high (for a rare earth) extinction coefficients. Determining the origin of these properties will require the isolation of more examples of other ligand systems for these Ln^2+^ ions. Although the electronic nature of these Ln^2+^ complexes may not be completely understood, it is clear they can function as multi-electron reductants that can provide four electrons from a single molecule. Hence, these complexes demonstrate a new approach using f element chemistry to multi-electron reducing systems, which are not very common.

## Supplementary Material

Supplementary information

Crystal structure data
