# Supplementary material for: Isolation of +2 rare earth metal ions with three anionic carbocyclic rings: bimetallic bis(cyclopentadienyl) reduced arene complexes of La2+ and Ce2+ are four electron reductants
Source: Chem Sci. 2015 Sep 21;6(12):7267–73. doi: 10.1039/c5sc02486b (PMC5947538; doi:10.1039/c5sc02486b)
Supplement: Supplementary file 1 [file SC-006-C5SC02486B-s001.pdf]

Electronic Supplementary Material (ESI) for Chemical Science.
This journal is © The Royal Society of Chemistry 2015

Electronic Supporting Information

Isolation of +2 Rare Earth Metal Ions with Three Anionic Carbocyclic Rings: Bimetallic Bis(cyclopentadienyl) Reduced Arene Complexes of La^{2+} and Ce^{2+} are Four Electron Reductants

Christopher M. Kotyk,[†] Megan E. Fieser,[†] Chad T. Palumbo,[†] Joseph W. Ziller,[†] Lucy E. Darago,[‡] Jeffrey R. Long,[‡]* Filipp Furche,[†]* and William J. Evans[†]*

[†]Department of Chemistry, University of California, Irvine, California 92697, United States

[‡]Department of Chemistry, University of California, Berkeley, California 94720, United States

Table of Contents

S1	Title page
S2	Table of Contents
S3-S5	Experimental Details
S6-S7	Magnetism of [K(2.2.2-cryptand)]$_2$[(Cp′$_2$Ln)$_2$(μ-η^6:η^6-C_6H_6)] (Ln = La, Ce).
S7-S8	Magnetic Measurements
S8-S13	Computational Details
S14-S37	X-ray Data Collection, Structure Solution and Refinement for [K(2.2.2-cryptand)]$_2$[(Cp′$_2$La)$_2$(μ-η^6:η^6-C_6H_6)], **2-La**.
S38-S39	X-ray Data Collection, Structure Solution and Refinement for [K(2.2.2-cryptand)]$_2$[(Cp′$_2$Ce)$_2$(μ-η^6:η^6-C_6H_6)], **2-Ce**.
S39	Definitions
S40	References

Experimental Details

The syntheses and manipulations described below were conducted under argon with rigorous exclusion of air and water using glovebox, vacuum line, and Schlenk techniques. Solvents were sparged with UHP argon (Praxair) and passed through columns containing Q-5 and molecular sieves before use. NMR solvents (Cambridge Isotope Laboratories) were dried over NaK/benzophenone, degassed by three freeze–pump–thaw cycles, and vacuum transferred prior to use. Anhydrous $Cp'_3La(THF)$,[1] Cp'_3Ln (Ln = Ce,[2] Pr,[3] Nd[4]), and KC_8[5] were prepared according to the literature. 2.2.2-Cryptand, (4,7,13,16,21,24-hexaoxa-1,10-diazabicyclo[8.8.8]hexacosane), (Aldrich) was placed under vacuum (10^{-3} Torr) for 12 h before use. ^{1}H NMR (500 MHz) and ^{13}C NMR (125 MHz) were obtained on a Bruker GN500 or CRYO500 MHz spectrometer at 298 K. IR samples were prepared as KBr pellets and the spectra were obtained on a Jasco FT/IR-4700 spectrometer. Elemental analyses were performed on a Perkin-Elmer 2400 Series II CHNS elemental analyzer.

[K(2.2.2-cryptand)]$_2$[(Cp′$_2$La)$_2$(μ-η^6:η^6-C_6H_6)], 2-La. In an argon-filled glovebox, benzene (10 mL) was added to a vial containing $Cp'_3La(THF)$, (156 mg, 0.251 mmol), 2.2.2-cryptand (189 mg, 0.502 mmol), and KC_8 (68 mg, 0.503 mmol). The mixture was allowed to stir vigorously for 4 hours. When stirring ceased, the suspension was allowed to settle and colorless benzene was pipetted out of the vial and discarded leaving behind a black oil. 2 mL of THF was added to the oil and stirred for 5 minutes before centrifugation for 10 min to remove graphite. The dark purple supernatant was filtered into a vial and chilled at −35 °C for an hour. The solution was layered with room temperature Et_2O (10 mL) and stored at −35 °C, producing dark purple crystals overnight (72 mg, 33 %). X-ray quality crystals were grown from a concentrated THF solution at −35 °C over 5 days. ^{1}H NMR (THF-d_8): δ 5.70 (s, CH_4SiMe_3,

8H), 5.62 (s, CH_4SiMe_3, 8H), 3.55 (s, OCH_2CH_2O, 24H), 3.50 (s, NCH_2CH_2O, 24H), 2.53 (s, NCH_2CH_2O, 24H), 2.04 (s, C_6H_6, 6H), 0.21 (s, $C_5H_4SiMe_3$, 27H). ^{13}C NMR (THF-d_8): δ 113.7 (CH_4SiMe_3), 109.1 (CH_4SiMe_3), 71.7 (OCH_2CH_2O), 69.6 (C_6H_6), 68.7 (NCH_2CH_2O), 55.4 (NCH_2CH_2O), 2.9 ($C_5H_4SiMe_3$). IR: 3068w, 2946s, 2883s, 2811s, 1478m, 1444m, 1385m, 1360s, 1354s, 1299m, 1259m, 1237m, 1179m, 1134s, 1104s, 1079s, 1037m, 950s, 933m, 904m, 831s, 749s, 732m, 689w, 678w, 668w, 634m. Anal. Calcd for $C_{74}H_{130}K_2La_2N_4O_{12}Si_4$•2THF, **2-La**: C, 52.38; H, 7.83; N, 2.98. Found: C, 52.52; H, 7.78; N, 3.00.

[K(2.2.2-cryptand)]$_2$[(Cp′$_2$Ce)$_2$(μ-η^6:η^6-C_6H_6)], 2-Ce. In an argon-filled glovebox, benzene (10 mL) was added to a vial containing Cp′$_3$Ce (175 mg, 0.387 mmol), 2.2.2-cryptand (298 mg, 0.791 mmol), and KC_8 (108 mg, 0.799 mmol). The mixture was allowed to stir vigorously for 4 hours. When stirring ceased, the suspension was allowed to settle and colorless benzene was pipetted out of the vial and discarded leaving behind a black oil. 2 mL of THF was added to the oil and stirred for 5 minutes before centrifugation for 10 min to remove graphite. The dark black supernatant was filtered into a vial and chilled at −35 °C for an hour. The solution was layered with room temperature Et_2O (10 mL) and stored at −35 °C, producing black crystals overnight (174 mg, 26 %). X-ray quality crystals were grown from a concentrated THF solution layered with Et_2O at −35 °C overnight. IR: 3068w, 2946s, 2882s, 2813s, 1478m, 1445m, 1388m, 1360s, 1354s, 1300m, 1258m, 1236m, 1179m, 1134s, 1104s, 1080s, 1036m, 950s, 932m, 904m, 828s, 749s, 730m, 716m, 678m, 634m, 628m. Anal. Calcd for $C_{74}H_{130}K_2Ce_2N_4O_{12}Si_4$•$C_6H_6$, **2-Ce**: C, 52.89; H, 7.55; N, 3.08. Found: C, 53.09; H, 7.87; N, 3.20.

Reaction of 2-La with Naphthalene. In an argon-filled glovebox, a solution of naphthalene (3 mg, 0.02 mmol) in THF (1 mL) was added to a stirred dark purple solution of **2-**

La (20 mg, 0.012 mmol) in THF (1 mL). The solution was stirred for 10 min, during which time the solution turned deep green. The solvent was removed under vacuum, and the resulting solids were washed with hexane and dried to give [K(2.2.2-cryptand)][$Cp'_2La(\eta^4-C_{10}H_8)$] as a deep green powder (19 mg, 86 %).

Reaction of 2-Ce with Naphthalene. In an argon-filled glovebox, a solution of naphthalene (11 mg, 0.08 mmol) in THF (1 mL) was added to a stirred dark purple solution of **2-Ce** (72 mg, 0.04 mmol) in THF (2 mL). The solution was stirred for 10 min, during which time the solution turned deep green. The solvent was removed under vacuum, and the resulting solids were washed with hexane and dried to give [K(2.2.2-cryptand)][$Cp'_2Ce(\eta^4-C_{10}H_8)$] as a deep green powder (72 mg, 91 %).

Magnetism of [K(2.2.2-cryptand)]$_2$[($Cp'_2Ln)_2(\mu-\eta^6:\eta^6-C_6H_6)$] (Ln = La, Ce).

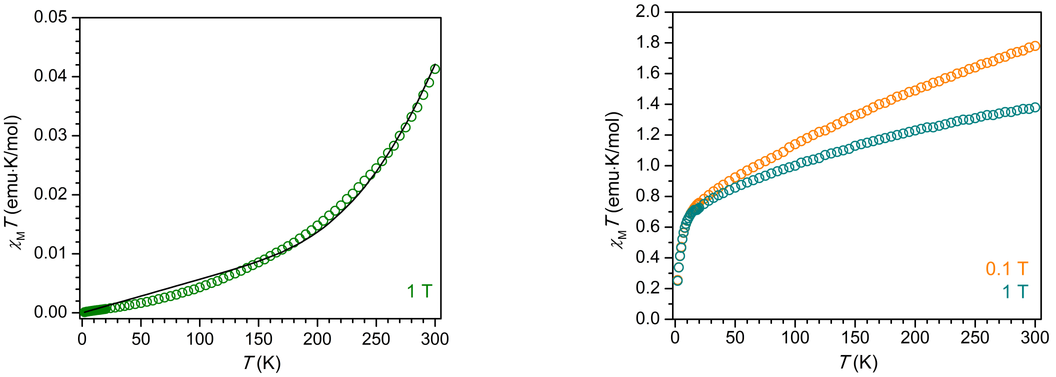

Fig. S.1 Variable-temperature magnetic susceptibility data collected for **2-La** under H_{dc} = 1 T (green). Simulation of the data using the model described in the text is represented by a solid black line. Variable-temperature magnetic susceptibility data collected for **2-Ce** under H_{dc} = 0.1 T (orange) and 1 T (blue).

The compound **2-La** exhibits diamagnetic behavior, with an extremely small (~10^{-2} emu·K/mol) magnetic susceptibility across the temperature range 2-300 K. The gradual increase in $\chi_M T$ with increasing temperature is likely due to a very small population of a triplet excited state or field-induced mixing with a triplet excited state. While the diamagnetism of **2-La** may at first seem indicative of a $La^{3+}/(C_6H_6)^{4-}$ electron configuration, we instead propose, in agreement with the crystallographic analysis, UV-Vis spectroscopy, and electronic structure calculations, that extremely strong magnetic exchange coupling between the two La^{2+} (d^1) centers and the $(C_6H_6)^{2-}$ diradical generates a well-isolated singlet ground state for **2-La**. Indeed, DFT calculations reveal the energy splitting between the ground state and first excited triplet state to be 12000 cm^{-1}, well over an order of magnitude higher than the thermal energy at 300 K. The magnetic data could also be reasonably well-simulated using the Hamiltonian $\hat{H} = -2J_{\mathrm{La-C6H6}}\left(\hat{S}_{\mathrm{La1}} \cdot \hat{S}_{\mathrm{C6H6}} + \hat{S}_{\mathrm{La2}} \cdot \hat{S}_{\mathrm{C6H6}}\right)$, where $J_{\mathrm{La-C6H6}}$ represents the coupling between the S = ½ La^{2+} centers and the S = 1 $(C_6H_6)^{2-}$ bridging unit, along with a temperature-independent paramagnetism contribution, χ_{TIP}. The best simulation was achieved using the values $J_{\mathrm{La-C6H6}} = -497$ cm^{-1} and χ_{TIP} = 0.000057 emu/mol (Figure X). Thus, the diamagnetism observed across the measured temperature range is also consistent with an assignment of $La^{2+}/(C_6H_6)^{2-}$ together with a pairwise exchange constant of $J_{\mathrm{La-C6H6}} < |-500$ $cm^{-1}|$.

The variable-temperature magnetic susceptibility of **2-Ce** alone also does not enable a definitive assignment of the oxidation states present. At 300 K under an applied magnetic field of 0.1 T, **2-Ce** exhibits a $\chi_M T$ product of 1.78 emu·K/mol, which then drops to 1.36 emu·K/mol under an increased applied magnetic field of 1 T. The field dependence of **2-Ce** is due to temperature-independent paramagnetism, which has been previously observed for both Ce^{2+} and

Ce^{3+} compounds.[6, 7] The expected $\chi_M T$ value at 300 K for a $Ce^{3+}/(C_6H_6)^{4-}$ configuration is 1.60 emu·K/mol. The situation for a Ce^{2+}-based electronic configuration is much more complicated, as even for a simple mononuclear Ce^{2+} complex the room temperature $\chi_M T$ value falls in between those expected for "uncoupled" and "coupled" $4f^1 5d^1$ configurations.[6] The LS coupling schemes for $4f^n 5d^1$ configurations detailed in ref. 1 can be used, in tandem with the assumption that at 300 K the S = 1 $(C_6H_6)^{2-}$ moiety is not magnetically coupled to the Ce^{2+} ions, to predict room temperature $\chi_M T$ values for **2-Ce**. The calculated $\chi_M T$ products at 300 K for **2-Ce** assuming "uncoupled" and "coupled" $4f^1 5d^1$ configurations plus an isolated S = 1 center are 3.36 emu·K/mol and 1.66 emu·K/mol, respectively. Clearly the former value does not match the $\chi_M T$ data observed for **2-Ce**. However, the latter value for the "coupled" scheme is quite close to the experimental $\chi_M T$ product for **2-Ce**. If magnetic coupling between the Ce^{2+} spins and the $(C_6H_6)^{2-}$ diradical is strong, as proposed above for **2-La**, the expected $\chi_M T$ product at 300 K for **2-Ce** will be even lower than 1.66 emu·K/mol, and thus closer to the observed value of 1.36 emu·K/mol at 1 T. If extremely strong d-π* magnetic coupling is present in **2-Ce**, the resulting magnetic behavior could even appear much like that of a standard dinuclear Ce^{3+} ($4f^1$) molecule. Therefore, discerning between the possible electronic configurations for **2-Ce**, $Ce^{3+}/(C_6H_6)^{4-}$ or $Ce^{2+}/(C_6H_6)^{2-}$, is not possible from the magnetic data alone.

Magnetic Measurements

Samples were prepared by adding crystalline powder of **2-La** (76.0 mg) and **2-Ce** (58.5 mg) to a 5 mm inner diameter quartz tube containing a raised quartz platform. Solid eicosane was added to cover the sample to prevent crystallite torqueing and provide good thermal contact

between the sample and the cryostat. The tubes were fitted with Teflon sealable adapters, evacuated on a Schlenk line, and flame-sealed under static vacuum. Following flame sealing, the solid eicosane was melted in a water bath held at 40 °C. Magnetic susceptibility measurements were performed using a Quantum Design MPMS2 SQUID magnetometer. Dc magnetic susceptibility measurements were collected in the temperature range 2-300 K under applied magnetic fields of 0.1 T and 1 T. Diamagnetic corrections were applied to the data using Pascal's constants to give $\chi_D = -0.00104808$ emu/mol (**2-La**), $\chi_D = -0.00104808$ emu/mol (**2-Ce**), and $\chi_D = -0.00024306$ emu/mol (eicosane).

Computational Details

Structural optimizations starting from the crystal data of [K(2.2.2-cryptand)]$_2$[(Cp'$_2$Ln)$_2$(μ-η^6:η^6-C_6H_6)], **2-La**, were carried out using density functional theory (DFT) for several possible electronic occupations to determine the ground state of the complex. Countercations were removed from the experimental crystal structures before the start of each optimization. Singlet, open shell singlet (spin unrestricted), and quintet (spin unrestricted) states were investigated for **2-La**. The hybrid meta-GGA functional of Tao, Perdew, Staroverov, and Scuseria, TPSSh,[8] was used in conjunction with double-zeta quality split-valence basis sets with polarization functions [def2-SV(P)][9] for all non-hydrogen, light atoms. Small quasi-relativistic effective core potentials[10] (ECPs) and triple-zeta quality basis sets (def2-TZVP)[11] were used for La. Vibrational frequencies[12] were computed for the optimized structures and were confirmed to be potential energy minima by the absence of imaginary modes.

In order to account for solvation effects, a second structural optimization was carried out using the continuum solvation model (COSMO).[13] The dielectric constant of THF (ε = 7.520)[14]

was used for the reported COSMO calculations since this was the most polar solvent used in the synthesis. Solvent effects have been shown to be important for Ln^{2+} systems because they screen the additional negative charge.[3, 15-18] The changes in bond length between the SV(P) optimized structures and those from a third optimization using TZVP basis sets for light atoms were typically smaller than 0.02 Å for the singlet state. All results were computed in C_1 symmetry, and ground state energies were converged to 10^{-7} a.u. using fine quadrature grids (at least size m4).[19] Molecular orbitals were plotted with a contour value of 0.05. All calculations were performed using the Turbomole quantum chemistry software.[20]

The results of the third optimization described above, using COSMO and TZVP basis sets for all atoms, indicated the singlet structure was lower in energy for **2-La**. This calculation also matched the crystal structure reasonably, although a La–$C(C_6H_6)$ bond length elongation of 0.05 Å was observed in comparison to the crystal structure. Calculations of the quintet state were not consistent between the SV(P) optimized structure and optimization using TZVP basis sets for light atoms, neither of which matched the crystal structure. Additional spin unrestricted single-point energy calculations of the singlet-triplet and singlet-quintet energy gaps were then performed on the optimized TPSSh singlet structure for **2-La**. The triplet state was found to be 30 kcal/mol (10500 cm^{-1}) higher and the quintet state was found to be 62 kcal/mol (21800 cm^{-1}) higher than the singlet ground state. Mulliken population analysis (MPA)[21] and natural population analysis (NPA)[22] were used to further understand the electronic properties of this complex.

Time dependent DFT (TDDFT) excitation energy calculations[23] of the first 90 excitations for **2-La** were computed from self-consistent orbitals. The excitation energy calculations were performed twice, once with the SV(P) basis set for light atoms and once with diffuse functions

added to the basis set (def2-SVPD)[24] for light atoms, while small core ECPs and TZVP basis sets were used for lanthanum. To simulate the UV-Vis spectra, a normalized Gaussian scaled by the predicted oscillator strength was centered at each molecular excitation, and a root mean square width of 0.20 eV was chosen to fit experiment.[25] The simulated spectrum with the SV(P) basis set for the light atoms was a much better fit to the experimental spectrum, and is therefore the simulated spectrum discussed in the manuscript. Representative excitations (in nm) and oscillator strengths in the length gauge (in a.u.) from each band, and dominant single-particle contributions for each transition are reported in Table S1. Transitions were analyzed with MPA, Table S2.[21]

Table S1. Electronic excitation summary for the dianion of **2-La**, computed using the TPSSh functional and SV(P) basis sets. Only pertinent excitations with oscillator strengths larger than 10^{-3} a.u. are included.

Compound	Wavelength	Oscillator Strength	Dominant Contributions		
	(nm)	(len)	occupied	virtual	% comp
**2-La**	694.01	0.014	183a	185a	63.1
			182a	184a	33.7
	616.87	0.082	183a	186a	49.7
			182a	184a	30.3
			183a	185a	19.5
	534.17	0.002	182a	186a	93.0
	527.82	0.493	183a	186a	46.7
			182a	184a	32.0
			183a	185a	12.5

482.05	0.004	182a	187a	82.7
		183a	190a	14.5
456.99	0.002	183a	189a	96.7
449.47	0.004	183a	190a	81.7
		182a	187a	15.6
440.28	0.138	182a	188a	92.9
436.29	0.042	183a	192a	79.9
		183a	191a	10.4
426.46	0.085	183a	191a	85.5
423.89	0.071	182a	189a	84.8
		183a	192a	10.0
422.72	0.022	182a	190a	87.1
418.88	0.033	183a	193a	93.0
406.70	0.028	182a	192a	57.1
		182a	191a	40.8
399.90	0.025	182a	191a	55.9
		182a	192a	41.2
390.91	0.032	182a	193a	98.8
378.34	0.001	183a	195a	84.8
		183a	194a	13.4
371.11	0.010	183a	197a	83.4
355.57	0.009	182a	195a	82.8
		182a	194a	13.0
350.48	0.033	182a	196a	49.8
		183a	199a	36.8
340.99	0.068	182a	198a	96.5
337.08	0.052	183a	203a	84.0
		182a	200a	12.2

334.59	0.109	182a	200a	59.6
		182a	201a	16.7
		183a	199a	10.2
326.93	0.012	183a	204a	94.2
326.09	0.017	182a	201a	77.4
		182a	200a	15.6
319.99	0.009	182a	202a	96.9
307.92	0.026	183a	206a	94.0
302.03	0.001	183a	209a	80.9

Table S2. Mulliken population analysis summary for the dianion in **2-La**. The % metal character indicates the total metal contribution (from both La centers combined) to the molecular orbital, the % d character indicates how much of the total orbital comes directly from the metal d orbitals.

Compound	Molecular Orbital	% Metal Character	% d Character
**2-La**	HOMO−1 (182a)	37	29
	HOMO (183a)	42	40
	LUMO (184a)	60	27
	LUMO+1 (185a)	72	18
	LUMO+2 (186a)	90	16
	LUMO+3 (187a)	50	46
	LUMO+4 (188a)	77	4
	LUMO+5	38	0

(189a)		
LUMO+6 (190a)	24	19
LUMO+7 (191a)	33	2
LUMO+8 (192a)	54	0
LUMO+9 (193a)	14	5
LUMO+10 (194a)	21	0
LUMO+11 (195a)	19	0
LUMO+12 (196a)	25	13
LUMO+13 (197a)	21	2
LUMO+14 (198a)	42	6
LUMO+15 (199a)	31	17
LUMO+16 (200a)	19	6
LUMO+17 (201a)	33	33
LUMO+18 (202a)	2	0
LUMO+19 (203a)	38	16
LUMO+22 (206a)	17	9
LUMO+25 (209a)	33	25

X-ray Data Collection, Structure Solution and Refinement for 2-La.

A purple crystal of approximate dimensions 0.077 x 0.175 x 0.299 mm was mounted on a glass fiber and transferred to a Bruker SMART APEX II diffractometer. The APEX2[26] program package was used to determine the unit-cell parameters and for data collection (60 sec/frame scan time for a sphere of diffraction data). The raw frame data was processed using SAINT[27] and SADABS[28] to yield the reflection data file. Subsequent calculations were carried out using the SHELXTL[29] program. The diffraction symmetry was *mmm* and the systematic absences were consistent with the orthorhombic space group *Pccn* that was later determined to be correct.

The structure was solved by direct methods and refined on F^2 by full-matrix least-squares techniques. The analytical scattering factors[30] for neutral atoms were used throughout the analysis. Hydrogen atoms were included using a riding model. There was one molecule of tetrahydrofuran and one-half molecule of benzene present per formula-unit. The benzene was located about an inversion center. Potassium atoms K(2) and K(3) were located on two-fold rotation axes.

At convergence, wR2 = 0.1305 and Goof = 1.211 for 933 variables refined against 18865 data (0.80Å), R1 = 0.0553 for those 16255 data with $I > 2.0\sigma(I)$.

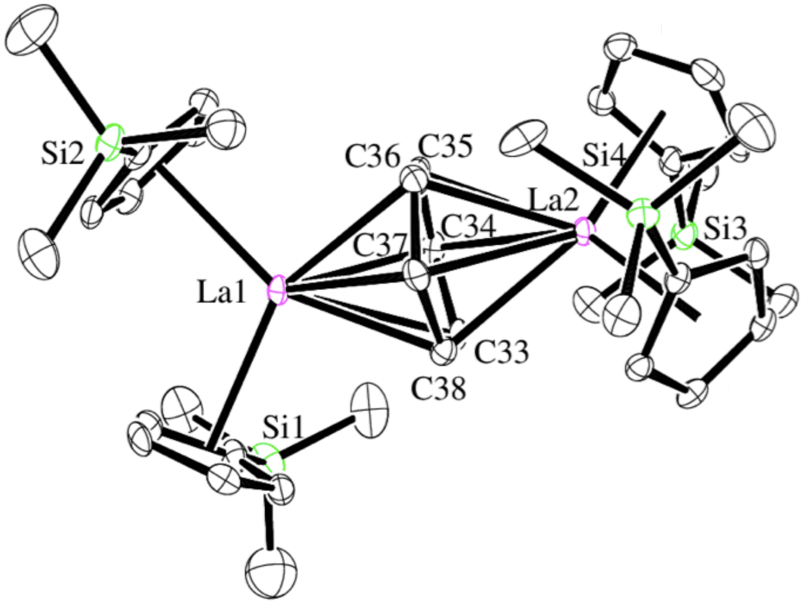

Fig. S.2 Molecular structure of the anion of [K(2.2.2-cryptand)]$_2$[(Cp′$_2$La)$_2$(μ-η^6:η^6-C_6H_6)], **2-La**. Thermal ellipsoids are drawn at the 50% probability level. Hydrogen atoms and co-crystallized benzene and THF were omitted for clarity.

Table S3. Crystal data and structure refinement for **2-La**.

Empirical formula	C_{74} H_{130} La_2 K_2 N_4 O_{12} Si_4 • 1.5(C_6H_6) • C_4H_8O	
Formula weight	1847.35	
Temperature	88(2) K	
Wavelength	0.71073 Å	
Crystal system	Orthorhombic	
Space group	*Pccn*	
Unit cell dimensions	a = 20.3261(11) Å	α = 90°.
	b = 54.668(3) Å	β = 90°.
	c = 16.5827(9) Å	γ = 90°.
Volume	18426.5(17) Å^3	
Z	8	
Density (calculated)	1.332 Mg/m^3	

Absorption coefficient	1.114 mm^{-1}
F(000)	7736
Crystal color	purple
Crystal size	0.299 x 0.175 x 0.077 mm^3
Theta range for data collection	1.069 to 26.372°
Index ranges	$-25 \leq h \leq 25$, $-68 \leq k \leq 68$, $-20 \leq l \leq 20$
Reflections collected	194473
Independent reflections	18865 [R(int) = 0.0505]
Completeness to theta = 25.500°	100.0 %
Absorption correction	Semi-empirical from equivalents
Max. and min. transmission	0.7457 and 0.6462
Refinement method	Full-matrix least-squares on F^2
Data / restraints / parameters	18865 / 0 / 933
Goodness-of-fit on F^2	1.211
Final R indices [I>2sigma(I) = 16255 data]	R1 = 0.0553, wR2 = 0.1258
R indices (all data, 0.80Å)	R1 = 0.0650, wR2 = 0.1305
Largest diff. peak and hole	2.510 and -1.474 e.$Å^{-3}$

Table S4. Bond lengths [Å] and angles [°] for **2-La**.

La(1)-Cnt1	2.692
La(1)-Cnt2	2.681
La(2)-Cnt3	2.687
La(2)-Cnt4	2.709
La(1)-C(34)	2.639(4)
La(1)-C(37)	2.641(5)
La(1)-C(38)	2.680(4)

La(1)-C(35)	2.694(5)
La(1)-C(33)	2.770(4)
La(1)-C(36)	2.777(5)
La(1)-C(1)	2.929(5)
La(1)-C(12)	2.929(5)
La(1)-C(11)	2.939(5)
La(1)-C(13)	2.943(5)
La(1)-C(9)	2.944(5)
La(1)-C(5)	2.945(5)
La(1)-C(10)	2.946(5)
La(1)-C(2)	2.946(5)
La(1)-C(4)	2.954(5)
La(1)-C(3)	2.967(5)
La(2)-C(36)	2.635(5)
La(2)-C(33)	2.637(5)
La(2)-C(38)	2.676(4)
La(2)-C(35)	2.683(4)
La(2)-C(37)	2.766(4)
La(2)-C(34)	2.776(5)
La(2)-C(26)	2.932(4)
La(2)-C(18)	2.937(5)
La(2)-C(25)	2.939(4)
La(2)-C(19)	2.942(5)
La(2)-C(17)	2.942(5)
La(2)-C(20)	2.944(4)
La(2)-C(21)	2.962(4)
La(2)-C(27)	2.967(4)
La(2)-C(29)	2.992(4)
La(2)-C(28)	2.996(4)
Si(1)-C(6)	1.832(8)
Si(1)-C(5)	1.862(5)
Si(1)-C(8)	1.868(6)
Si(1)-C(7)	1.903(6)
Si(2)-C(13)	1.852(5)
Si(2)-C(14)	1.871(6)
Si(2)-C(16)	1.872(6)

Si(2)-C(15)	1.885(6)
Si(3)-C(21)	1.850(5)
Si(3)-C(22)	1.860(5)
Si(3)-C(23)	1.865(5)
Si(3)-C(24)	1.885(5)
Si(4)-C(29)	1.850(5)
Si(4)-C(32)	1.865(5)
Si(4)-C(30)	1.870(6)
Si(4)-C(31)	1.884(5)
C(1)-C(2)	1.409(7)
C(1)-C(5)	1.426(7)
C(2)-C(3)	1.415(7)
C(3)-C(4)	1.400(7)
C(4)-C(5)	1.420(7)
C(9)-C(10)	1.409(7)
C(9)-C(13)	1.419(7)
C(10)-C(11)	1.413(7)
C(11)-C(12)	1.410(7)
C(12)-C(13)	1.441(7)
C(17)-C(18)	1.402(7)
C(17)-C(21)	1.441(7)
C(18)-C(19)	1.420(7)
C(19)-C(20)	1.406(7)
C(20)-C(21)	1.426(6)
C(25)-C(26)	1.411(6)
C(25)-C(29)	1.425(6)
C(26)-C(27)	1.411(7)
C(27)-C(28)	1.415(6)
C(28)-C(29)	1.433(6)
C(33)-C(38)	1.454(6)
C(33)-C(34)	1.457(6)
C(34)-C(35)	1.448(7)
C(35)-C(36)	1.446(6)
C(36)-C(37)	1.459(6)
C(37)-C(38)	1.456(6)
K(1)-O(6)	2.717(3)

K(1)-O(2)	2.814(3)
K(1)-O(3)	2.822(3)
K(1)-O(1)	2.827(3)
K(1)-O(5)	2.873(4)
K(1)-O(4)	2.878(3)
K(1)-N(1)	3.008(4)
K(1)-N(2)	3.019(4)
K(2)-O(9)	2.819(4)
K(2)-O(9)#1	2.819(4)
K(2)-O(8)	2.831(4)
K(2)-O(8)#1	2.831(4)
K(2)-O(7)#1	2.855(4)
K(2)-O(7)	2.855(4)
K(2)-N(3)#1	2.962(4)
K(2)-N(3)	2.962(4)
K(3)-O(11)	2.826(3)
K(3)-O(11)#2	2.827(3)
K(3)-O(12)#2	2.833(4)
K(3)-O(12)	2.833(4)
K(3)-O(10)	2.836(4)
K(3)-O(10)#2	2.837(4)
K(3)-N(4)	2.917(4)
K(3)-N(4)#2	2.917(4)
O(1)-C(40)	1.427(6)
O(1)-C(41)	1.428(6)
O(2)-C(42)	1.424(6)
O(2)-C(43)	1.426(6)
O(3)-C(46)	1.422(7)
O(3)-C(47)	1.426(6)
O(4)-C(48)	1.424(6)
O(4)-C(49)	1.430(6)
O(5)-C(53)	1.423(6)
O(5)-C(52)	1.428(6)
O(6)-C(55)	1.423(6)
O(6)-C(54)	1.425(6)
O(7)-C(58)	1.416(7)

O(7)-C(59)	1.423(7)
O(8)-C(60)	1.412(7)
O(8)-C(61)	1.431(7)
O(9)-C(64)	1.402(9)
O(9)-C(63)	1.436(7)
O(10)-C(68)	1.426(7)
O(10)-C(67)	1.430(7)
O(11)-C(69)	1.425(6)
O(11)-C(70)	1.428(6)
O(12)-C(73)	1.409(8)
O(12)-C(72)	1.441(7)
N(1)-C(45)	1.465(7)
N(1)-C(51)	1.470(6)
N(1)-C(39)	1.473(6)
N(2)-C(44)	1.472(6)
N(2)-C(50)	1.477(7)
N(2)-C(56)	1.479(6)
N(3)-C(62)	1.462(7)
N(3)-C(57)	1.469(8)
N(3)-C(65)	1.476(8)
N(4)-C(66)	1.466(8)
N(4)-C(71)	1.468(7)
N(4)-C(74)	1.475(7)
C(39)-C(40)	1.509(8)
C(41)-C(42)	1.497(7)
C(43)-C(44)	1.508(7)
C(45)-C(46)	1.516(8)
C(47)-C(48)	1.490(8)
C(49)-C(50)	1.506(8)
C(51)-C(52)	1.504(7)
C(53)-C(54)	1.499(7)
C(55)-C(56)	1.508(8)
C(57)-C(58)	1.509(9)
C(59)-C(60)	1.475(9)
C(61)-C(65)#1	1.501(8)
C(62)-C(63)	1.512(10)

C(64)-C(64)#1	1.496(14)
C(65)-C(61)#1	1.501(8)
C(66)-C(67)	1.515(9)
C(68)-C(69)	1.497(8)
C(70)-C(74)#2	1.511(8)
C(71)-C(72)	1.484(10)
C(73)-C(73)#2	1.517(14)
C(74)-C(70)#2	1.510(8)
C(75)-C(76)	1.379(8)
C(75)-C(77)#3	1.385(8)
C(76)-C(77)	1.391(8)
C(77)-C(75)#3	1.385(8)
O(13)-C(81)	1.447(11)
O(13)-C(78)	1.460(12)
C(78)-C(79)	1.508(14)
C(79)-C(80)	1.651(15)
C(80)-C(81)	1.446(13)

Cnt1-La(1)-Cnt2	111.5
Cnt1-La(1)-Cnt5	125.1
Cnt2-La(1)-Cnt5	123.4
Cnt3-La(1)-Cnt4	112.6
Cnt3-La(1)-Cnt5	123.0
Cnt4-La(1)-Cnt5	124.5
Cnt1-La(1)-C(33)	98.1
Cnt1-La(1)-C(34)	121.8
Cnt1-La(1)-C(35)	152.1
Cnt1-La(1)-C(36)	146.5
Cnt1-La(1)-C(37)	115.5
Cnt1-La(1)-C(38)	95.1
Cnt2-La(1)-C(33)	144.6
Cnt2-La(1)-C(35)	113.5
Cnt2-La(1)-C(35)	93.5
Cnt2-La(1)-C(36)	97.0
Cnt2-La(1)-C(37)	121.1
Cnt2-La(1)-C(38)	151.2

Cnt3-La(2)-C(33)	120.9
Cnt3-La(2)-C(34)	96.7
Cnt3-La(2)-C(35)	92.9
Cnt3-La(2)-C(36)	113.0
Cnt3-La(2)-C(37)	144.1
Cnt3-La(2)-C(38)	151.0
Cnt4-La(2)-C(33)	114.3
Cnt4-La(2)-C(34)	145.3
Cnt4-La(2)-C(35)	152.2
Cnt4-La(2)-C(36)	122.2
Cnt4-La(2)-C(37)	97.8
Cnt4-La(2)-C(38)	94.2
C(34)-La(1)-C(37)	67.29(14)
C(34)-La(1)-C(38)	55.95(14)
C(37)-La(1)-C(38)	31.74(14)
C(34)-La(1)-C(35)	31.50(14)
C(37)-La(1)-C(35)	55.77(13)
C(38)-La(1)-C(35)	63.90(13)
C(34)-La(1)-C(33)	31.13(14)
C(37)-La(1)-C(33)	56.21(14)
C(38)-La(1)-C(33)	30.90(13)
C(35)-La(1)-C(33)	54.29(14)
C(34)-La(1)-C(36)	55.84(14)
C(37)-La(1)-C(36)	31.10(13)
C(38)-La(1)-C(36)	54.40(14)
C(35)-La(1)-C(36)	30.61(13)
C(33)-La(1)-C(36)	63.61(14)
C(34)-La(1)-C(1)	101.16(14)
C(37)-La(1)-C(1)	97.35(14)
C(38)-La(1)-C(1)	72.26(14)
C(35)-La(1)-C(1)	128.85(14)
C(33)-La(1)-C(1)	74.56(14)
C(36)-La(1)-C(1)	126.24(14)
C(34)-La(1)-C(12)	97.40(14)
C(37)-La(1)-C(12)	98.88(14)
C(38)-La(1)-C(12)	127.26(14)

C(35)-La(1)-C(12)	71.83(13)
C(33)-La(1)-C(12)	125.92(13)
C(36)-La(1)-C(12)	72.89(14)
C(1)-La(1)-C(12)	158.97(13)
C(34)-La(1)-C(11)	90.72(14)
C(37)-La(1)-C(11)	121.04(14)
C(38)-La(1)-C(11)	140.59(14)
C(35)-La(1)-C(11)	76.71(14)
C(33)-La(1)-C(11)	121.69(14)
C(36)-La(1)-C(11)	90.84(14)
C(1)-La(1)-C(11)	141.36(14)
C(12)-La(1)-C(11)	27.79(14)
C(34)-La(1)-C(13)	125.23(14)
C(37)-La(1)-C(13)	103.10(14)
C(38)-La(1)-C(13)	134.71(13)
C(35)-La(1)-C(13)	96.86(14)
C(33)-La(1)-C(13)	150.09(14)
C(36)-La(1)-C(13)	87.58(14)
C(1)-La(1)-C(13)	133.48(14)
C(12)-La(1)-C(13)	28.41(13)
C(11)-La(1)-C(13)	46.39(13)
C(34)-La(1)-C(9)	136.26(14)
C(37)-La(1)-C(9)	129.82(14)
C(38)-La(1)-C(9)	159.60(13)
C(35)-La(1)-C(9)	117.05(13)
C(33)-La(1)-C(9)	166.83(13)
C(36)-La(1)-C(9)	114.83(14)
C(1)-La(1)-C(9)	113.30(13)
C(12)-La(1)-C(9)	45.68(13)
C(11)-La(1)-C(9)	45.55(13)
C(13)-La(1)-C(9)	27.90(13)
C(34)-La(1)-C(5)	101.81(14)
C(37)-La(1)-C(5)	123.93(14)
C(38)-La(1)-C(5)	95.06(13)
C(35)-La(1)-C(5)	133.25(14)
C(33)-La(1)-C(5)	85.67(13)

C(36)-La(1)-C(5)	148.19(13)
C(1)-La(1)-C(5)	28.10(13)
C(12)-La(1)-C(5)	137.07(14)
C(11)-La(1)-C(5)	113.62(14)
C(13)-La(1)-C(5)	123.88(13)
C(9)-La(1)-C(5)	96.96(13)
C(34)-La(1)-C(10)	112.30(14)
C(37)-La(1)-C(10)	144.63(14)
C(38)-La(1)-C(10)	167.60(14)
C(35)-La(1)-C(10)	104.14(14)
C(33)-La(1)-C(10)	139.80(14)
C(36)-La(1)-C(10)	117.14(14)
C(1)-La(1)-C(10)	116.59(14)
C(12)-La(1)-C(10)	45.80(14)
C(11)-La(1)-C(10)	27.78(14)
C(13)-La(1)-C(10)	46.26(13)
C(9)-La(1)-C(10)	27.68(13)
C(5)-La(1)-C(10)	91.29(14)
C(34)-La(1)-C(2)	125.18(14)
C(37)-La(1)-C(2)	93.82(13)
C(38)-La(1)-C(2)	80.61(14)
C(35)-La(1)-C(2)	144.50(14)
C(33)-La(1)-C(2)	95.04(14)
C(36)-La(1)-C(2)	124.59(13)
C(1)-La(1)-C(2)	27.75(13)
C(12)-La(1)-C(2)	137.12(13)
C(11)-La(1)-C(2)	138.78(14)
C(13)-La(1)-C(2)	108.76(14)
C(9)-La(1)-C(2)	96.05(13)
C(5)-La(1)-C(2)	46.08(13)
C(10)-La(1)-C(2)	111.31(14)
C(34)-La(1)-C(4)	127.11(14)
C(37)-La(1)-C(4)	138.89(14)
C(38)-La(1)-C(4)	117.73(14)
C(35)-La(1)-C(4)	156.18(14)
C(33)-La(1)-C(4)	113.45(14)

C(36)-La(1)-C(4)	169.93(13)
C(1)-La(1)-C(4)	45.48(13)
C(12)-La(1)-C(4)	114.42(14)
C(11)-La(1)-C(4)	98.58(14)
C(13)-La(1)-C(4)	96.31(14)
C(9)-La(1)-C(4)	70.37(14)
C(5)-La(1)-C(4)	27.86(13)
C(10)-La(1)-C(4)	71.68(14)
C(2)-La(1)-C(4)	45.34(13)
C(34)-La(1)-C(3)	145.74(14)
C(37)-La(1)-C(3)	117.10(14)
C(38)-La(1)-C(3)	108.26(14)
C(35)-La(1)-C(3)	172.07(14)
C(33)-La(1)-C(3)	119.76(14)
C(36)-La(1)-C(3)	144.30(13)
C(1)-La(1)-C(3)	45.54(13)
C(12)-La(1)-C(3)	114.32(13)
C(11)-La(1)-C(3)	111.10(14)
C(13)-La(1)-C(3)	88.07(13)
C(9)-La(1)-C(3)	69.78(13)
C(5)-La(1)-C(3)	45.84(13)
C(10)-La(1)-C(3)	83.76(14)
C(2)-La(1)-C(3)	27.68(13)
C(4)-La(1)-C(3)	27.35(13)
C(36)-La(2)-C(33)	67.35(14)
C(36)-La(2)-C(38)	56.04(14)
C(33)-La(2)-C(38)	31.76(13)
C(36)-La(2)-C(35)	31.54(14)
C(33)-La(2)-C(35)	55.91(14)
C(38)-La(2)-C(35)	64.09(14)
C(36)-La(2)-C(37)	31.22(13)
C(33)-La(2)-C(37)	56.31(14)
C(38)-La(2)-C(37)	30.98(13)
C(35)-La(2)-C(37)	54.50(13)
C(36)-La(2)-C(34)	55.89(14)
C(33)-La(2)-C(34)	31.09(14)

C(38)-La(2)-C(34)	54.43(13)
C(35)-La(2)-C(34)	30.71(14)
C(37)-La(2)-C(34)	63.73(13)
C(36)-La(2)-C(26)	126.51(13)
C(33)-La(2)-C(26)	93.03(14)
C(38)-La(2)-C(26)	80.76(13)
C(35)-La(2)-C(26)	144.79(14)
C(37)-La(2)-C(26)	96.04(13)
C(34)-La(2)-C(26)	123.62(14)
C(36)-La(2)-C(18)	113.31(15)
C(33)-La(2)-C(18)	144.81(14)
C(38)-La(2)-C(18)	168.68(14)
C(35)-La(2)-C(18)	104.91(14)
C(37)-La(2)-C(18)	140.78(14)
C(34)-La(2)-C(18)	117.68(14)
C(26)-La(2)-C(18)	110.30(14)
C(36)-La(2)-C(25)	102.07(13)
C(33)-La(2)-C(25)	95.52(13)
C(38)-La(2)-C(25)	71.08(13)
C(35)-La(2)-C(25)	129.11(13)
C(37)-La(2)-C(25)	74.69(13)
C(34)-La(2)-C(25)	124.79(13)
C(26)-La(2)-C(25)	27.81(12)
C(18)-La(2)-C(25)	117.52(13)
C(36)-La(2)-C(19)	90.53(14)
C(33)-La(2)-C(19)	122.18(14)
C(38)-La(2)-C(19)	141.17(14)
C(35)-La(2)-C(19)	77.08(14)
C(37)-La(2)-C(19)	121.50(14)
C(34)-La(2)-C(19)	91.89(14)
C(26)-La(2)-C(19)	138.05(14)
C(18)-La(2)-C(19)	27.96(14)
C(25)-La(2)-C(19)	142.14(13)
C(36)-La(2)-C(17)	136.30(14)
C(33)-La(2)-C(17)	128.56(14)
C(38)-La(2)-C(17)	158.44(14)

C(35)-La(2)-C(17)	116.35(14)
C(37)-La(2)-C(17)	167.21(14)
C(34)-La(2)-C(17)	113.73(13)
C(26)-La(2)-C(17)	95.44(13)
C(18)-La(2)-C(17)	27.59(14)
C(25)-La(2)-C(17)	114.24(13)
C(19)-La(2)-C(17)	45.81(14)
C(36)-La(2)-C(20)	96.03(14)
C(33)-La(2)-C(20)	99.53(14)
C(38)-La(2)-C(20)	127.46(13)
C(35)-La(2)-C(20)	70.85(13)
C(37)-La(2)-C(20)	124.99(13)
C(34)-La(2)-C(20)	73.03(13)
C(26)-La(2)-C(20)	137.10(13)
C(18)-La(2)-C(20)	45.54(13)
C(25)-La(2)-C(20)	159.88(13)
C(19)-La(2)-C(20)	27.63(14)
C(17)-La(2)-C(20)	45.65(13)
C(36)-La(2)-C(21)	123.00(13)
C(33)-La(2)-C(21)	102.08(14)
C(38)-La(2)-C(21)	133.81(13)
C(35)-La(2)-C(21)	94.55(13)
C(37)-La(2)-C(21)	148.11(13)
C(34)-La(2)-C(21)	85.78(13)
C(26)-La(2)-C(21)	109.31(13)
C(18)-La(2)-C(21)	46.14(14)
C(25)-La(2)-C(21)	134.93(13)
C(19)-La(2)-C(21)	46.21(13)
C(17)-La(2)-C(21)	28.25(13)
C(20)-La(2)-C(21)	27.94(13)
C(36)-La(2)-C(27)	146.21(13)
C(33)-La(2)-C(27)	117.05(14)
C(38)-La(2)-C(27)	108.43(13)
C(35)-La(2)-C(27)	172.36(14)
C(37)-La(2)-C(27)	120.06(13)
C(34)-La(2)-C(27)	144.07(13)

C(26)-La(2)-C(27)	27.67(13)
C(18)-La(2)-C(27)	82.63(14)
C(25)-La(2)-C(27)	45.49(13)
C(19)-La(2)-C(27)	110.39(13)
C(17)-La(2)-C(27)	69.91(13)
C(20)-La(2)-C(27)	114.94(13)
C(21)-La(2)-C(27)	89.87(13)
C(36)-La(2)-C(29)	101.75(13)
C(33)-La(2)-C(29)	121.47(13)
C(38)-La(2)-C(29)	92.85(13)
C(35)-La(2)-C(29)	133.29(13)
C(37)-La(2)-C(29)	84.36(13)
C(34)-La(2)-C(29)	146.41(13)
C(26)-La(2)-C(29)	45.96(12)
C(18)-La(2)-C(29)	93.38(13)
C(25)-La(2)-C(29)	27.79(12)
C(19)-La(2)-C(29)	114.92(13)
C(17)-La(2)-C(29)	99.71(13)
C(20)-La(2)-C(29)	138.91(13)
C(21)-La(2)-C(29)	127.20(13)
C(27)-La(2)-C(29)	45.80(12)
C(36)-La(2)-C(28)	126.39(13)
C(33)-La(2)-C(28)	137.68(13)
C(38)-La(2)-C(28)	116.22(13)
C(35)-La(2)-C(28)	155.92(14)
C(37)-La(2)-C(28)	112.05(13)
C(34)-La(2)-C(28)	168.76(13)
C(26)-La(2)-C(28)	45.30(13)
C(18)-La(2)-C(28)	72.50(13)
C(25)-La(2)-C(28)	45.19(12)
C(19)-La(2)-C(28)	98.97(13)
C(17)-La(2)-C(28)	72.76(13)
C(20)-La(2)-C(28)	115.99(13)
C(21)-La(2)-C(28)	99.63(12)
C(27)-La(2)-C(28)	27.44(12)
C(29)-La(2)-C(28)	27.70(12)

C(6)-Si(1)-C(5)	112.2(3)
C(6)-Si(1)-C(8)	107.6(4)
C(5)-Si(1)-C(8)	115.4(2)
C(6)-Si(1)-C(7)	105.4(4)
C(5)-Si(1)-C(7)	108.5(3)
C(8)-Si(1)-C(7)	107.1(3)
C(13)-Si(2)-C(14)	110.6(3)
C(13)-Si(2)-C(16)	112.3(2)
C(14)-Si(2)-C(16)	107.6(3)
C(13)-Si(2)-C(15)	109.4(2)
C(14)-Si(2)-C(15)	108.1(3)
C(16)-Si(2)-C(15)	108.7(3)
C(21)-Si(3)-C(22)	108.8(2)
C(21)-Si(3)-C(23)	114.0(2)
C(22)-Si(3)-C(23)	109.4(3)
C(21)-Si(3)-C(24)	111.5(2)
C(22)-Si(3)-C(24)	106.2(3)
C(23)-Si(3)-C(24)	106.5(3)
C(29)-Si(4)-C(32)	116.6(2)
C(29)-Si(4)-C(30)	108.1(2)
C(32)-Si(4)-C(30)	108.3(3)
C(29)-Si(4)-C(31)	109.3(2)
C(32)-Si(4)-C(31)	105.3(2)
C(30)-Si(4)-C(31)	109.0(3)
C(2)-C(1)-C(5)	108.8(4)
C(2)-C(1)-La(1)	76.8(3)
C(5)-C(1)-La(1)	76.6(3)
C(1)-C(2)-C(3)	107.8(4)
C(1)-C(2)-La(1)	75.4(3)
C(3)-C(2)-La(1)	77.0(3)
C(4)-C(3)-C(2)	107.8(4)
C(4)-C(3)-La(1)	75.8(3)
C(2)-C(3)-La(1)	75.3(3)
C(3)-C(4)-C(5)	109.5(4)
C(3)-C(4)-La(1)	76.9(3)
C(5)-C(4)-La(1)	75.7(3)

C(4)-C(5)-C(1)	106.1(4)
C(4)-C(5)-Si(1)	124.8(4)
C(1)-C(5)-Si(1)	128.8(4)
C(4)-C(5)-La(1)	76.4(3)
C(1)-C(5)-La(1)	75.3(3)
Si(1)-C(5)-La(1)	119.3(2)
C(10)-C(9)-C(13)	109.8(4)
C(10)-C(9)-La(1)	76.2(3)
C(13)-C(9)-La(1)	76.0(3)
C(9)-C(10)-C(11)	107.6(4)
C(9)-C(10)-La(1)	76.1(3)
C(11)-C(10)-La(1)	75.9(3)
C(12)-C(11)-C(10)	108.2(4)
C(12)-C(11)-La(1)	75.7(3)
C(10)-C(11)-La(1)	76.4(3)
C(11)-C(12)-C(13)	108.7(4)
C(11)-C(12)-La(1)	76.5(3)
C(13)-C(12)-La(1)	76.3(3)
C(9)-C(13)-C(12)	105.7(4)
C(9)-C(13)-Si(2)	126.7(4)
C(12)-C(13)-Si(2)	127.5(4)
C(9)-C(13)-La(1)	76.1(3)
C(12)-C(13)-La(1)	75.3(3)
Si(2)-C(13)-La(1)	117.9(2)
C(18)-C(17)-C(21)	108.8(4)
C(18)-C(17)-La(2)	76.0(3)
C(21)-C(17)-La(2)	76.7(3)
C(17)-C(18)-C(19)	108.5(4)
C(17)-C(18)-La(2)	76.4(3)
C(19)-C(18)-La(2)	76.2(3)
C(20)-C(19)-C(18)	107.3(4)
C(20)-C(19)-La(2)	76.3(3)
C(18)-C(19)-La(2)	75.8(3)
C(19)-C(20)-C(21)	109.8(4)
C(19)-C(20)-La(2)	76.1(3)
C(21)-C(20)-La(2)	76.7(3)

C(20)-C(21)-C(17)	105.6(4)
C(20)-C(21)-Si(3)	128.3(4)
C(17)-C(21)-Si(3)	125.9(4)
C(20)-C(21)-La(2)	75.3(3)
C(17)-C(21)-La(2)	75.1(3)
Si(3)-C(21)-La(2)	119.5(2)
C(26)-C(25)-C(29)	109.3(4)
C(26)-C(25)-La(2)	75.8(3)
C(29)-C(25)-La(2)	78.2(2)
C(25)-C(26)-C(27)	108.0(4)
C(25)-C(26)-La(2)	76.4(3)
C(27)-C(26)-La(2)	77.5(3)
C(26)-C(27)-C(28)	107.8(4)
C(26)-C(27)-La(2)	74.8(3)
C(28)-C(27)-La(2)	77.4(3)
C(27)-C(28)-C(29)	109.0(4)
C(27)-C(28)-La(2)	75.2(2)
C(29)-C(28)-La(2)	76.0(2)
C(25)-C(29)-C(28)	105.9(4)
C(25)-C(29)-Si(4)	126.5(3)
C(28)-C(29)-Si(4)	127.1(3)
C(25)-C(29)-La(2)	74.0(2)
C(28)-C(29)-La(2)	76.3(2)
Si(4)-C(29)-La(2)	121.67(19)
C(38)-C(33)-C(34)	118.0(4)
C(38)-C(33)-La(2)	75.6(3)
C(34)-C(33)-La(2)	79.7(3)
C(38)-C(33)-La(1)	71.1(2)
C(34)-C(33)-La(1)	69.4(2)
La(2)-C(33)-La(1)	114.60(17)
C(35)-C(34)-C(33)	118.3(4)
C(35)-C(34)-La(1)	76.4(3)
C(33)-C(34)-La(1)	79.4(3)
C(35)-C(34)-La(2)	71.1(2)
C(33)-C(34)-La(2)	69.2(2)
La(1)-C(34)-La(2)	114.36(16)

C(36)-C(35)-C(34)	122.6(4)
C(36)-C(35)-La(2)	72.4(2)
C(34)-C(35)-La(2)	78.2(3)
C(36)-C(35)-La(1)	77.9(3)
C(34)-C(35)-La(1)	72.2(3)
La(2)-C(35)-La(1)	115.66(17)
C(35)-C(36)-C(37)	118.5(4)
C(35)-C(36)-La(2)	76.0(3)
C(37)-C(36)-La(2)	79.3(3)
C(35)-C(36)-La(1)	71.5(3)
C(37)-C(36)-La(1)	69.3(2)
La(2)-C(36)-La(1)	114.44(17)
C(38)-C(37)-C(36)	117.8(4)
C(38)-C(37)-La(1)	75.6(2)
C(36)-C(37)-La(1)	79.6(3)
C(38)-C(37)-La(2)	71.1(2)
C(36)-C(37)-La(2)	69.5(2)
La(1)-C(37)-La(2)	114.61(16)
C(33)-C(38)-C(37)	122.5(4)
C(33)-C(38)-La(2)	72.6(2)
C(37)-C(38)-La(2)	77.9(2)
C(33)-C(38)-La(1)	78.0(3)
C(37)-C(38)-La(1)	72.7(2)
La(2)-C(38)-La(1)	116.36(17)
O(6)-K(1)-O(2)	91.59(11)
O(6)-K(1)-O(3)	126.43(11)
O(2)-K(1)-O(3)	137.39(11)
O(6)-K(1)-O(1)	127.97(11)
O(2)-K(1)-O(1)	60.28(10)
O(3)-K(1)-O(1)	99.98(11)
O(6)-K(1)-O(5)	60.24(10)
O(2)-K(1)-O(5)	115.99(11)
O(3)-K(1)-O(5)	100.98(10)
O(1)-K(1)-O(5)	91.98(10)
O(6)-K(1)-O(4)	102.55(11)
O(2)-K(1)-O(4)	98.19(10)

O(3)-K(1)-O(4)	58.99(10)
O(1)-K(1)-O(4)	122.79(10)
O(5)-K(1)-O(4)	140.83(11)
O(6)-K(1)-N(1)	118.98(11)
O(2)-K(1)-N(1)	121.55(11)
O(3)-K(1)-N(1)	60.31(11)
O(1)-K(1)-N(1)	61.74(10)
O(5)-K(1)-N(1)	59.30(10)
O(4)-K(1)-N(1)	118.77(11)
O(6)-K(1)-N(2)	62.03(11)
O(2)-K(1)-N(2)	58.30(10)
O(3)-K(1)-N(2)	119.00(11)
O(1)-K(1)-N(2)	117.96(11)
O(5)-K(1)-N(2)	121.74(11)
O(4)-K(1)-N(2)	60.44(11)
N(1)-K(1)-N(2)	178.96(12)
O(9)-K(2)-O(9)#1	60.56(18)
O(9)-K(2)-O(8)	118.62(13)
O(9)#1-K(2)-O(8)	99.89(12)
O(9)-K(2)-O(8)#1	99.89(12)
O(9)#1-K(2)-O(8)#1	118.62(13)
O(8)-K(2)-O(8)#1	135.73(17)
O(9)-K(2)-O(7)#1	141.27(14)
O(9)#1-K(2)-O(7)#1	99.34(13)
O(8)-K(2)-O(7)#1	96.19(12)
O(8)#1-K(2)-O(7)#1	58.74(11)
O(9)-K(2)-O(7)	99.34(13)
O(9)#1-K(2)-O(7)	141.27(14)
O(8)-K(2)-O(7)	58.74(11)
O(8)#1-K(2)-O(7)	96.19(12)
O(7)#1-K(2)-O(7)	113.87(17)
O(9)-K(2)-N(3)#1	119.74(14)
O(9)#1-K(2)-N(3)#1	60.65(13)
O(8)-K(2)-N(3)#1	61.22(12)
O(8)#1-K(2)-N(3)#1	118.61(13)
O(7)#1-K(2)-N(3)#1	61.18(13)

O(7)-K(2)-N(3)#1	118.58(12)
O(9)-K(2)-N(3)	60.65(13)
O(9)#1-K(2)-N(3)	119.75(14)
O(8)-K(2)-N(3)	118.61(13)
O(8)#1-K(2)-N(3)	61.22(12)
O(7)#1-K(2)-N(3)	118.58(12)
O(7)-K(2)-N(3)	61.17(13)
N(3)#1-K(2)-N(3)	179.6(2)
O(11)-K(3)-O(11)#2	137.95(16)
O(11)-K(3)-O(12)#2	100.21(11)
O(11)#2-K(3)-O(12)#2	116.54(12)
O(11)-K(3)-O(12)	116.54(11)
O(11)#2-K(3)-O(12)	100.21(11)
O(12)#2-K(3)-O(12)	59.12(19)
O(11)-K(3)-O(10)	58.93(10)
O(11)#2-K(3)-O(10)	96.25(11)
O(12)#2-K(3)-O(10)	143.14(12)
O(12)-K(3)-O(10)	100.78(12)
O(11)-K(3)-O(10)#2	96.25(11)
O(11)#2-K(3)-O(10)#2	58.94(10)
O(12)#2-K(3)-O(10)#2	100.78(12)
O(12)-K(3)-O(10)#2	143.14(12)
O(10)-K(3)-O(10)#2	110.85(16)
O(11)-K(3)-N(4)	118.56(12)
O(11)#2-K(3)-N(4)	61.36(11)
O(12)#2-K(3)-N(4)	118.71(14)
O(12)-K(3)-N(4)	61.47(13)
O(10)-K(3)-N(4)	61.63(12)
O(10)#2-K(3)-N(4)	118.25(12)
O(11)-K(3)-N(4)#2	61.37(11)
O(11)#2-K(3)-N(4)#2	118.56(12)
O(12)#2-K(3)-N(4)#2	61.47(13)
O(12)-K(3)-N(4)#2	118.71(14)
O(10)-K(3)-N(4)#2	118.25(12)
O(10)#2-K(3)-N(4)#2	61.63(12)
N(4)-K(3)-N(4)#2	179.8(2)

C(40)-O(1)-C(41)	110.5(4)
C(40)-O(1)-K(1)	115.1(3)
C(41)-O(1)-K(1)	108.7(3)
C(42)-O(2)-C(43)	112.5(4)
C(42)-O(2)-K(1)	117.7(3)
C(43)-O(2)-K(1)	120.3(3)
C(46)-O(3)-C(47)	111.4(4)
C(46)-O(3)-K(1)	119.0(3)
C(47)-O(3)-K(1)	117.8(3)
C(48)-O(4)-C(49)	111.3(4)
C(48)-O(4)-K(1)	113.4(3)
C(49)-O(4)-K(1)	116.1(3)
C(53)-O(5)-C(52)	110.7(4)
C(53)-O(5)-K(1)	113.2(3)
C(52)-O(5)-K(1)	115.8(3)
C(55)-O(6)-C(54)	110.8(4)
C(55)-O(6)-K(1)	119.3(3)
C(54)-O(6)-K(1)	117.2(3)
C(58)-O(7)-C(59)	112.1(5)
C(58)-O(7)-K(2)	115.8(4)
C(59)-O(7)-K(2)	116.5(3)
C(60)-O(8)-C(61)	110.2(4)
C(60)-O(8)-K(2)	117.4(3)
C(61)-O(8)-K(2)	118.1(3)
C(64)-O(9)-C(63)	110.6(5)
C(64)-O(9)-K(2)	114.3(4)
C(63)-O(9)-K(2)	118.1(3)
C(68)-O(10)-C(67)	110.4(4)
C(68)-O(10)-K(3)	117.1(3)
C(67)-O(10)-K(3)	116.9(3)
C(69)-O(11)-C(70)	110.7(4)
C(69)-O(11)-K(3)	117.4(3)
C(70)-O(11)-K(3)	119.3(3)
C(73)-O(12)-C(72)	111.6(5)
C(73)-O(12)-K(3)	117.0(4)
C(72)-O(12)-K(3)	117.6(3)

C(45)-N(1)-C(51)	108.1(4)
C(45)-N(1)-C(39)	111.5(4)
C(51)-N(1)-C(39)	109.5(4)
C(45)-N(1)-K(1)	109.1(3)
C(51)-N(1)-K(1)	112.0(3)
C(39)-N(1)-K(1)	106.7(3)
C(44)-N(2)-C(50)	108.0(4)
C(44)-N(2)-C(56)	110.2(4)
C(50)-N(2)-C(56)	110.7(4)
C(44)-N(2)-K(1)	113.1(3)
C(50)-N(2)-K(1)	110.1(3)
C(56)-N(2)-K(1)	104.8(3)
C(62)-N(3)-C(57)	109.8(5)
C(62)-N(3)-C(65)	109.2(5)
C(57)-N(3)-C(65)	110.5(5)
C(62)-N(3)-K(2)	110.2(3)
C(57)-N(3)-K(2)	109.3(3)
C(65)-N(3)-K(2)	107.8(3)
C(66)-N(4)-C(71)	110.4(5)
C(66)-N(4)-C(74)	110.5(5)
C(71)-N(4)-C(74)	110.0(5)
C(66)-N(4)-K(3)	109.4(3)
C(71)-N(4)-K(3)	108.4(3)
C(74)-N(4)-K(3)	108.2(3)
N(1)-C(39)-C(40)	113.4(4)
O(1)-C(40)-C(39)	109.9(4)
O(1)-C(41)-C(42)	109.9(4)
O(2)-C(42)-C(41)	108.6(4)
O(2)-C(43)-C(44)	108.0(4)
N(2)-C(44)-C(43)	112.3(4)
N(1)-C(45)-C(46)	114.7(4)
O(3)-C(46)-C(45)	108.5(4)
O(3)-C(47)-C(48)	108.7(4)
O(4)-C(48)-C(47)	109.1(4)
O(4)-C(49)-C(50)	110.3(4)
N(2)-C(50)-C(49)	114.6(4)

N(1)-C(51)-C(52)	113.9(4)
O(5)-C(52)-C(51)	108.2(4)
O(5)-C(53)-C(54)	108.4(4)
O(6)-C(54)-C(53)	109.2(5)
O(6)-C(55)-C(56)	109.9(4)
N(2)-C(56)-C(55)	113.5(4)
N(3)-C(57)-C(58)	113.9(5)
O(7)-C(58)-C(57)	110.1(5)
O(7)-C(59)-C(60)	110.9(5)
O(8)-C(60)-C(59)	109.9(5)
O(8)-C(61)-C(65)#1	109.3(5)
N(3)-C(62)-C(63)	113.5(5)
O(9)-C(63)-C(62)	109.0(5)
O(9)-C(64)-C(64)#1	110.7(7)
N(3)-C(65)-C(61)#1	114.4(5)
N(4)-C(66)-C(67)	114.1(5)
O(10)-C(67)-C(66)	109.5(5)
O(10)-C(68)-C(69)	109.7(5)
O(11)-C(69)-C(68)	108.9(5)
O(11)-C(70)-C(74)#2	109.5(4)
N(4)-C(71)-C(72)	115.1(5)
O(12)-C(72)-C(71)	109.5(5)
O(12)-C(73)-C(73)#2	109.6(5)
N(4)-C(74)-C(70)#2	114.5(5)
C(76)-C(75)-C(77)#3	120.3(6)
C(75)-C(76)-C(77)	119.4(5)
C(75)#3-C(77)-C(76)	120.2(5)
C(81)-O(13)-C(78)	105.7(7)
O(13)-C(78)-C(79)	108.4(8)
C(78)-C(79)-C(80)	101.8(9)
C(81)-C(80)-C(79)	103.9(9)
C(80)-C(81)-O(13)	111.8(8)

X-ray Structure Solution and Refinement for 2-Ce.

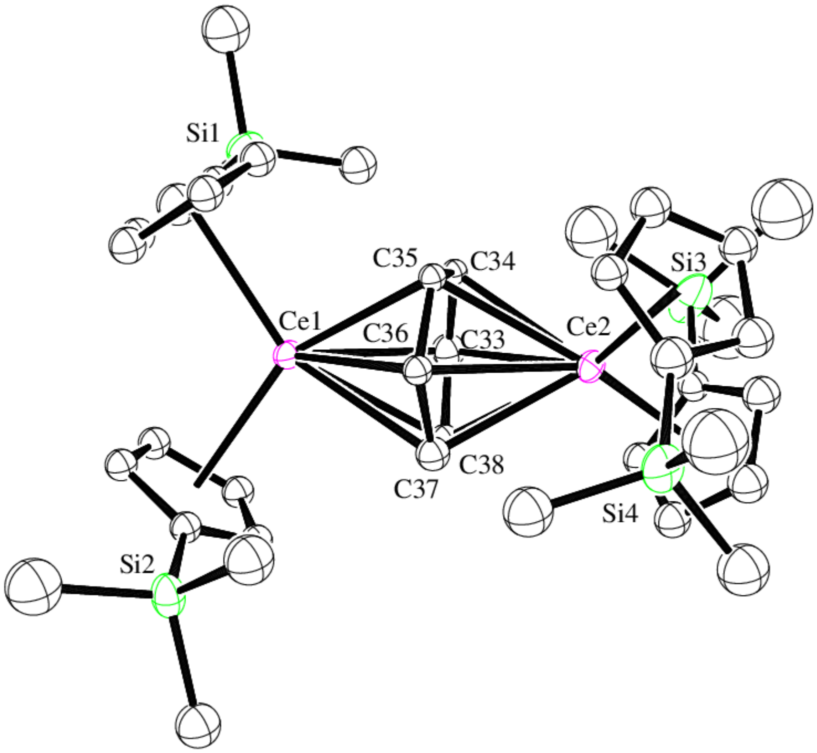

Fig. S.3 Connectivity-only molecular structure of the anion of [K(2.2.2-cryptand)]$_2$[(Cp′$_2$Ce)$_2$(μ-η^6:η^6-C_6H_6)], **2-Ce**. Thermal ellipsoids are drawn at the 50% probability level. Hydrogen atoms and co-crystallized benzene were omitted for clarity.

Table S5. Crystal data and structure refinement for **2-Ce**.

Empirical formula	C_{74} H_{130} Ce_2 K_2 N_4 O_{12} Si_4 • 1.5(C_6H_6)
Formula weight	1855.77
Temperature	138(2) K
Wavelength	0.71073 Å
Crystal system	Monoclinic
Space group	P2$_1$/c

Unit cell dimensions	a = 27.776(6) Å	α = 90°.
	b = 20.229(4) Å	β = 100.194(3)°.
	c = 16.486(4) Å	γ = 90°.
Volume	9116(3) Å^3	
Z	4	
Density (calculated)	1.352 Mg/m^3	
Absorption coefficient	1.187 mm^{-1}	
F(000)	3884	
Crystal color	black	
Crystal size	0.233 x 0.153 x 0.139 mm^3	

Definitions.

$wR2 = [\Sigma[w(F_o^2-F_c^2)^2] / \Sigma[w(F_o^2)^2]]^{1/2}$

$R1 = \Sigma||F_o|-|F_c|| / \Sigma|F_o|$

Goof = $S = [\Sigma[w(F_o^2-F_c^2)^2] / (n-p)]^{1/2}$ where n is the number of reflections and p is the total number of parameters refined.

The thermal ellipsoid plots are shown at the 50% probability level.

References

1. J. K. Peterson, M. R. MacDonald, J. W. Ziller and W. J. Evans, *Organometallics*, 2013, **32**, 2625-2631.
2. S. D. Stults, R. A. Andersen and A. Zalkin, *Organometallics*, 1990, **9**, 115–122.
3. M. R. MacDonald, J. E. Bates, J. W. Ziller, F. Furche and W. J. Evans, *J. Am. Chem. Soc.*, 2013, **135**, 9857-9868.
4. S. G. Minasian, J. L. Krinsky, J. D. Rinehart, R. Copping, T. Tyliszczak, M. Janousch, D. K. Shuh and J. Arnold, *J. Am. Chem. Soc.*, 2009, **131**, 13767-13783.
5. D. E. Bergbreiter and J. M. Killough, *J. Am. Chem. Soc.*, 1978, **100**, 2126-2134.
6. K. R. Meihaus, M. E. Fieser, J. F. Corbey, W. J. Evans and J. R. Long, *J. Am. Chem. Soc.*, 2015, **137**, 9855-9860.
7. M. D. Walter, C. H. Booth, W. W. Lukens and R. A. Andersen, *Organometallics*, 2009, **28**, 698-707.
8. V. N. Staroverov, G. E. Scuseria, J. Tao and J. P. Perdew, *J. Chem. Phys.*, 2003, **119**, 12129-12137.
9. A. Schafer, H. Horn and R. Ahlrichs, *J. Chem. Phys.*, 1992, **97**, 2571-2577.
10. D. Andrae, U. Hauessermann, M. Dolg, H. Stoll and H. Preuss, *Theor. Chim. Acta*, 1990, **77**, 123-141.
11. F. Weigend and R. Ahlrichs, *Phys. Chem. Chem. Phys.*, 2005, **7**, 3297-3305.
12. P. Deglmann, K. May, F. Furche and R. Ahlrichs, *Chem. Phys. Lett.*, 2004, **384**, 103-107.
13. A. Klamt and G. J. Schueuermann, *Chem. Soc., Perkin Trans. 2*, 1993, 799.
14. *CRC Handbook of Chemistry and Physics*, 81st edn., CRC Press, Boca Raton, FL, 2008.
15. M. R. MacDonald, J. E. Bates, M. E. Fieser, J. W. Ziller, F. Furche and W. J. Evans, *J. Am. Chem. Soc.*, 2012, **134**, 8420-8423.
16. M. R. MacDonald, M. E. Fieser, J. E. Bates, J. W. Ziller, F. Furche and W. J. Evans, *J. Am. Chem. Soc.*, 2013, **135**, 13310-13313.
17. M. E. Fieser, M. R. MacDonald, B. T. Krull, J. E. Bates, J. W. Ziller, F. Furche and W. J. Evans, *J. Am. Chem. Soc.*, 2015, **137**, 369-382.
18. R. R. Langeslay, M. E. Fieser, J. W. Ziller, F. Furche and W. J. Evans, *Chem. Sci.*, 2015, **6**, 517-521.
19. O. Treutler and R. Ahlrichs, *J. Chem. Phys.*, 1995, **102**, 346-354.
20. F. Furche, R. Ahlrichs, C. Hattig, W. Klopper, M. Sierka and F. Weigend, *WIREs Comput. Mol. Sci.*, 2014, **4**, 91-100.
21. R. S. Mulliken, *J. Chem. Phys.*, 1955, **23**, 1833-1840.
22. A. E. Reed, R. B. Weinstock and F. Weinhold, *J. Chem. Phys.*, 1985, **83**, 735-746.
23. R. Bauernschmitt and R. Ahlrichs, *Chem. Phys. Lett.*, 1996, **256**, 454-464.
24. D. Rappoport and F. Furche, *J. Chem. Phys.*, 2010, **133**, 134105-134101.
25. A. Brown, C. M. Kemp and S. F. Mason, *J. Chem. Soc.*, 1971, **A**, 751-755.
26. APEX2 Version 2014.9-0, Bruker AXS, Inc.; Madison, WI 2014.
27. SAINT Version 8.34a, Bruker AXS, Inc.; Madison, WI 2013.
28. G. M. Sheldrick, SADABS, Version 2014/5, Bruker AXS, Inc.; Madison, WI 2014.
29. G. M. Sheldrick, SHELXTL, Version 2014/7, Bruker AXS, Inc.; Madison, WI 2014.
30. International Tables for Crystallography 1992, Vol. C., Dordrecht: Kluwer Academic Publishers
